# A Social Support Just-in-Time Adaptive Intervention for Individuals With Depressive Symptoms: Feasibility Study With a Microrandomized Trial Design

**DOI:** 10.2196/74103

**Published:** 2025-08-26

**Authors:** Timon Elmer, Markus Wolf, Evelien Snippe, Urte Scholz

**Affiliations:** 1 Applied Social and Health Psychology Department of Psychology University of Zurich Zurich Switzerland; 2 Clinical Psychology with Focus on Psychotherapy Research Department of Psychology University of Zurich Zurich Switzerland; 3 Population Research Center University of Zurich Zurich Switzerland; 4 Department of Developmental Psychology University of Groningen Groningen The Netherlands

**Keywords:** just-in-time adaptive intervention, digital health intervention, feasibility, ecological momentary assessment, depression, mental health, help seeking, social support, mixed methods

## Abstract

**Background:**

Just-in-time adaptive interventions (JITAIs) aim to provide psychological support during critical moments in daily life.

**Objective:**

This preregistered study aims to evaluate the feasibility of a social support JITAI for individuals with subclinical and clinical levels of depressive symptoms awaiting psychotherapy. Triggered by ecological momentary assessment (EMA) reports, the intervention encouraged participants to activate their (digital) social support networks.

**Methods:**

A total of 25 participants completed 2689 EMA surveys and received 377 JITAIs over an 18-day intervention period, including a microrandomized trial, to compare 4 strategies to trigger an intervention: fixed cutoff points of distress variables, personalized thresholds (through Shewhart control charts) of distress variables, momentary support need, and no intervention.

**Results:**

The results showed high feasibility, with participants completing 85.37% (2689/3150) of the EMA surveys, exhibiting a low study-related attrition rate (7%; total attrition rate was 17%), and reporting minimal technical issues. Engagement and perceived helpfulness were heterogeneous and moderate, with participants seeking support in one-third of the instances after an intervention was triggered instances. JITAIs triggered by self-reported need for support were rated as more appropriately timed, helpful, and effective for promoting support-seeking behavior compared to those based on distress indicators, despite being triggered less frequently. Barriers, such as time constraints and perceived unavailability of support providers, likely affected support-seeking behavior, as indicated by additional qualitative analyses. Exploratory effectiveness analyses indicated Cohen *d* effect sizes between 0.06 and 0.14 in reducing distress after JITAIs were received.

**Conclusions:**

The findings of this study demonstrate that a social support JITAI is feasible to implement, with high compliance and minimal technical issues. However, further research is needed to evaluate the JITAI’s effectiveness and optimize trigger strategies in addressing individual needs for and barriers to engagement.

## Introduction

### Background

Mental health problems, such as depression, are becoming increasingly prevalent in today’s societies [1,2]. Despite the resulting need for mental health care systems, many countries are struggling to meet the demand for treatment. A large number of individuals around the globe report that their mental health needs are not sufficiently met [3-5] or are left untreated [6]. One reason for this is restricted access to mental health care because of long waiting times for outpatient psychotherapeutic treatment [7-9]. For example, depending on the area in the United States, the average waiting time for outpatient psychotherapeutic treatment is estimated to be between 19 days and 3 months [10-12]. In Germany, for example, the waiting time is estimated to be 16 weeks [13]. Long waiting times can have serious public health consequences. A delay in receiving psychotherapeutic treatment can exacerbate mental health conditions and may prolong distress [14,15].

One promising way to support individuals during this waiting period in coping with, for example, depressive symptoms is through digital mental health interventions delivered via the smartphone [11]. Digital mental health interventions have low entry barriers, can be cost-effective, and have the potential to reach a wide range of individuals, including those reluctant to seek psychotherapy due to stigma [16,17]. Examples of digital mental health interventions aiming to alleviate depressive symptoms include mindfulness training, self-monitoring reports, or elements of cognitive behavioral therapy (CBT) [18,19]. However, these static interventions often do not take into account the dynamic emotional experiences of participants’ daily lives, which could be used to determine optimal intervention time points.

The particularly promising forms of digital intervention in this context are just-in-time adaptive interventions (JITAIs) [20]. These interventions use sensor data or responses from repeated smartphone-based surveys, such as the experience sampling method or ecological momentary assessments (EMAs) [21-23], to trigger timely and contextually relevant interventions. JITAIs are designed to provide support precisely when and where it is needed most in daily life settings, allowing them to reach vulnerable individuals in critical moments [20,24]. Therefore, they represent a powerful approach that allows for real-time and personalized delivery of psychological interventions [25,26]. For instance, interventions can be triggered at critical moments when a particular individual is experiencing high levels of negative affect, stress, or rumination [27,28].

Despite the potential of smartphone-based JITAIs, current implementations often overlook one of the smartphone’s most obvious functions: connecting individuals to their social support networks. Social support is conceptualized as “a social network’s provision of psychological and material resources intended to benefit an individual’s ability to cope with stress” [29]. Because of this coping functionality, the availability of social support networks is a key determinant of mental health and a protective factor against the development of depression [30-32]. Socially supportive relationships protect from social isolation and its adverse effects on mental and physical health [29,33]. Seeking social support can be a major hurdle, especially for individuals experiencing depressive symptoms [34,35]. However, to the best of our knowledge, no mental health JITAI exists that aims to aid participants in seeking social support during vulnerable moments. Thus, there is a need to explore how JITAIs can be used to help individuals activate their (digital) social support networks.

### When Is “Just-in-Time”?

When designing a JITAI, a critical factor for its effectiveness is ensuring that it is provided at moments in participants’ daily lives when they need support and are receptive to it [20,36]. However, identifying these moments is challenging and requires a balance between accurately detecting distress and ensuring timely delivery. The decision rules that trigger the interventions thus need to be systematically studied when designing a JITAI, as these are assumed to affect proximal outcomes, such as intervention engagement [20].

Initially, interventions were triggered based on predefined, fixed cutoff points, such as scoring 4 or more on a 7-point negative affect scale [27,28]. While these cutoff points can capture general patterns, they fail to account for individual differences in what constitutes a “high” or “low” score. What may be normal for one person could represent significant distress for another. Moreover, the cutoff points do not consider natural fluctuations in individuals’ experiences over time, limiting their ability to identify meaningful deviations from a person’s usual state.

A newer approach that addresses these challenges is the use of *statistical process control* (SPC) methods, specifically *Shewhart control charts* (SCCs), to personalize the timing of interventions [37]. SPC charts track personal variability in key psychological indicators (eg, distress levels) and can be used to identify when someone deviates from their usual emotional state [37]. As interventions are triggered based on real-time changes, this method makes the timing more personalized.

However, a completely different approach may be effective as well, namely directly asking participants about their need for social support at any given moment. Many definitions of social support highlight the anticipation or mobilization of help from others during times of need, typically characterized by elevated stress [38]. Thus, assessing support need can serve as a pragmatic proxy for vulnerability and aligns with the theoretical accounts, emphasizing that support is most effective when individuals need it and are able to express their needs [39]. Hence, we assume that asking individuals directly about their current need for support may offer a more accurate and tailored strategy than relying solely on fixed cutoff points or inferred dynamics of distress variables. This assumption was preregistered on the Open Science Framework (OSF) [40].

### The Social Support JITAI

With the aim of helping individuals activate their (digital) social support networks, we designed a social support JITAI based on current best practice and research on social support. The intervention we proposed is designed to facilitate social support through JITAIs by sending prompts and reminders to connect with identified support figures during moments of distress, based on real-time data and responses gathered through EMAs. We identify moments of distress by assessing (fluctuations in) self-reported negative affect, stress, loneliness, and rumination. On the basis of a microrandomized controlled trial design [41] at each EMA assessment moment, it is randomly determined which condition the current assessment falls under: (1) after a participant’s distress level surpasses a fixed cutoff point (condition 1), (2) when the distress level surpasses a critical threshold based on SCCs (condition 2) [42], or (3) when participants indicate that they need support (condition 3). In condition 4, no intervention is triggered. In other words, at each EMA time point, participants were randomized to 1 of the 4 intervention-triggering conditions, making each assessment a distinct randomization unit in the microrandomized trial design. If triggered, the intervention includes prompts and messages designed to encourage participants to reach out to their (digital) social networks for support. Specifically, in line with the JITAI framework [20], when a vulnerable state is detected, the intervention is triggered. In the first step, participants are encouraged to reflect on what type of social support they would find helpful. This reflection is intended to activate problem-solving and coping strategies [43]. In the next step, participants are shown a list of past interaction partners, thereby highlighting potentially available support resources. Because states of vulnerability may be accompanied by narrowed thinking, seeing a list of possible support contacts may help broaden participants’ awareness of their social environment [44,45]. Finally, the JITAI encourages participants to seek social support from the available support provider in a way that is helpful for them, which is important for effective support seeking [39]. Participants were not limited to digital channels; they could choose to call, message, video chat, or meet in person based on their own preference and situational constraints. A detailed description of the social support JITAI can be found in the Methods section and Multimedia Appendix 1 and on the project’s OSF repository [40].

In line with the skilled support framework [46] and the optimal matching theory [47], this JITAI assumes that when social support is sought in the right moment, from the right person, and in the right way, it can help individuals to cope with distress-related adversity. Receiving social support in critical moments can help individuals by facilitating the feeling of belongingness, activating self-worth, receiving practical aid, developing coping strategies, and providing opportunities to express and regulate emotions or reinforcing positive behavior, thereby contributing to getting out of dysfunctional, depressed states [48,49]. Findings from correlational studies capturing social support behaviors and affective responses in daily life indeed suggest that higher received social support in crucial moments within everyday life contributes to a reduction of felt distress [50-52]. Furthermore, studies on social prescribing, a process where health care professionals refer patients to nonclinical social support interventions, may be effective in improving mental health [53,54]. However, no mental health JITAI has made use of the social support process.

### This Study

This study aims to address the gap in understanding the feasibility, acceptability, and initial intervention effects of a social support JITAI among individuals seeking psychotherapy. Feasibility studies are crucial, as they provide valuable insights into how well an intervention works in a real-world setting before proceeding to larger-scale trials. They help identify potential facilitators of and barriers to implementation, ensuring that the intervention is practical and effective [55,56]. In addition, high compliance rates and valid, high-quality data are essential for the JITAI to be triggered at appropriate moments, ensuring its effectiveness in real-world settings.

The first aim of this study is to examine various feasibility and proximal outcomes, specifically how participants report on (1) appropriateness of the interventions’ timing, (2) helpfulness, (3) behavior adoption (ie, whether they seek social support), (4) intervention engagement, (5) burden, (6) technical functioning, and (7) negative effects of the social support JITAI in general and specifically of the different triggering conditions. We expect that participants rate these aspects of feasibility and proximal outcomes in general as high. Other feasibility outcomes include the compliance rate with the EMA surveys and the data quality, measured through the rate of careless responses.

Second, we aim to evaluate which of the applied triggering strategies (ie, decision rules) is rated highest by the participants with regard to the perceived appropriate timing, helpfulness, and JITAI behavior adoption. We expect these outcomes to be the highest when the intervention is triggered after participants indicate they need support (condition 3), followed by interventions triggered based on fixed cutoff points (condition 1) or the SPC method (condition 2), as preregistered [40].

Third, we aim to explore the intervention effects of the social support JITAI on self-reported social support sought and momentary distress. We expect that participants are more likely to have sought social support and show a larger reduction in distress variables (negative affect, stress, loneliness, and rumination) between T (previous EMA time point; EMA time point that triggered the JITAI) and T+1 (subsequent EMA time point) after a JITAI (conditions 1, 2, or 3) compared to when no JITAI is triggered (condition 4).

## Methods

### Participants

Participants were individuals seeking outpatient psychotherapy who reported subclinical and clinical levels of depressive symptoms on the Beck Depression Inventory-II (BDI-II) with scores more than 9, indicating at least minimal depressive symptoms [57,58]. All participants were actively seeking outpatient psychotherapy at the time of study enrollment. While some were reported to be on waiting lists, many Swiss psychotherapists do not maintain explicit waiting lists; thus, some of the participants were seeking psychotherapy while not being on a waiting list. Other inclusion criteria were owning a smartphone and micro randomized providing informed consent. Exclusion criteria included individuals with a scheduled psychotherapy session within 4 weeks, suicidal ideation (values >2 on BDI-II item 9), manic symptoms (determined by the Mood Disorder Questionnaire) [59,60], or aged <18 or >70 years. Inclusion and exclusion criteria were assessed with a screening questionnaire.

Our sample consisted of 25 individuals. Of these, 22 (88%) self-identified as women and 3 (12%) identified as men. No other gender identification categories were reported. The mean age was 35.12 (SD 11.42) years. Most (n=21, 84%) participants self-reported their ethnicity as White. A detailed report of the demographic variables (including the level of education, employment status, living situation, and number of children) can be found in Table S1 in Multimedia Appendix 1.

### Procedure

All participants were led through the same procedure. Participants interested in this study, either by referral from psychotherapists who had to reject or put new clients on a waiting list or through our social media posts, were directed to a screening questionnaire. If eligible, participants were invited to an in-person meeting in which participants received information about the study (refer to the information sheet on OSF [40]), signed the informed consent form, completed a baseline survey assessing demographic information and depressive symptoms, installed the m-Path smartphone app for EMA surveys and JITAI delivery, and took part in a semistructured qualitative interview about social support seeking and smartphone-based interventions. Subsequently, EMA surveys were administered over a 21-day period using the m-Path software [61]. During this period, participants received 6 daily EMA surveys (randomly prompted between 8 AM and 10 AM, 10 AM and noon, noon and 2 PM, 2 PM and 4 PM, 4 PM and 6 PM, and 6 PM and 8 PM). After 3 days, JITAIs were additionally delivered through the m-Path app. The details on the intervention procedure are described in the Intervention section. Supplementary to the 6 EMA surveys, participants received an end-of-the-day survey via m-Path every evening (between 8 PM and 9 PM) and every 7 days (between 9 PM and 11 PM). After the 21-day EMA period, participants were again invited for a qualitative interview on their experiences during the study and a debriefing.

### Ethical Considerations

The study was approved by the ethics committee of the faculty of arts and sciences of the University of Zurich (23.04.11). All 25 participants provided informed consent. Participants were compensated with CHF 160 to 250 (US $197 to US $308), depending on their participation rate in the surveys delivered through the m-Path app (CHF 160 corresponded to 50%; CHF 250 corresponded to 100%). Ten days into the study, participants received information about their personal participation rate and were motivated to sustain or increase it by reminding them of their compensation.

### Measures

#### Distress Variables

The distress variables measured through EMAs included negative affect, stress, loneliness, and rumination. The original item formulations in German can be found on OSF [40]. In this study, we present their English translations. Negative affect was measured with a single item “to what extent do you experience negative emotions at the moment?” rated on a 7-point Likert scale ranging from 1 (I do not experience negative emotions) to 7 (very negative) [62]. Stress was measured with the item “I feel stressed at the moment” rated on a 7-point Likert scale ranging from 1 (disagree completely) to 7 (agree completely). Loneliness was measured with the item “I feel lonely at the moment” rated on a 7-point Likert scale ranging from 1 (disagree completely) to 7 (agree completely). Rumination was measured with the item “I realize that I am thinking the same negative thoughts over and over again” rated on a 7-point Likert scale ranging from 1 (disagree completely) to 7 (agree completely) [63].

#### Support Need

Support need was measured with the item “would it help you right now to talk to someone about your worries or negative feelings?”—which could be rated either as “yes,” “no, it would not help me,” or “I have no worries or negative feelings.”

#### Feasibility and Proximal Outcome Measures

A range of feasibility outcome measures was assessed using postintervention, evening, and weekly survey questions.

#### Postintervention Questions

The 2 questions mentioned subsequently were prompted at the beginning of the subsequent EMA survey after the intervention was triggered. They were embedded at the start of the next completed EMA survey, regardless of whether one or more scheduled EMAs had been missed in between. In our analyses, we used only those instances where a follow-up EMA was actually completed. JITAI ratings without a subsequent EMA were excluded from the analyses. Furthermore, no EMA lags that spanned overnight were considered. The appropriateness of the timing of the intervention was measured with the item “the last time the app made me aware that I could seek social support, it was just at the right time” rated on the 7-point Likert scale ranging from 1 (disagree completely) to 7 (agree completely). Whether the participant adopted the social support intervention was measured with the question “did you ask others for support because of the app’s advice?” which could be rated as “yes” or “no.”

#### Evening Questions

The perceived helpfulness of the intervention was measured with the item “the app’s instructions for activating social support were helpful” rated on a 7-point Likert scale ranging from 1 (disagree completely) to 7 (agree completely).

#### Weekly Questions

The intervention engagement was measured with the question “I have asked others for social support because of the app advice” on a 7-point Likert scale with the same anchors as earlier. The burden of completing the EMA surveys was also measured on a 7-point Likert scale with the question “I don’t mind completing the questionnaires for another week.” Technical problems were measured with the question “I had technical problems using the m-Path app,” also on a 7-point Likert scale. Negative effects of using the app were measured with the question “answering the questionnaires of this study has worsened my mental health” and the question “asking my social contacts for help has worsened my mental health.” These questions were rated on a 7-point Likert scale ranging from 1 (disagree completely) to 7 (agree completely).

#### Other Outcome Measures

Data quality was assessed with the attention check question “to ensure data quality, please select the value 5 for this question” on a 7-point Likert scale. This item was presented with a probability of 1 out of 7 to reduce the likelihood of participants learning when this item appears. Support seeking was measured with the question “I have asked someone for social support (since the last m-path questionnaire)” rated on a 7-point Likert scale ranging from 1 (disagree completely) to 7 (agree completely).

#### Baseline Survey

All demographic variables reported in Table S1 in Multimedia Appendix 1 were measured during the baseline survey. For detailed item formulations, refer to the OSF repository of this project [40].

### Intervention

#### Overview

The intervention designed in this study aimed to support individuals in activating their social support networks during critical moments of distress. When the JITAI was triggered, participants received personalized prompts on their smartphones through the m-Path app, encouraging them to reach out to members of their social network for support. The intervention included three core components: (1) specifying the type of support needed, (2) identifying who within the available network could provide this type of social support, and (3) receiving 1 of the 6 evidence-based strategies to facilitate effective support seeking. These strategies, including scholarly references, are described in detail on the OSF and aimed to encourage participants to clearly articulate their feelings and needs, reframe the situation, express gratitude, foster support reciprocation, and to diversify support providers [39,46,64,65]. Intervention delivery was randomized into 4 triggering conditions with a microrandomized trial design [41]. Participants were randomly assigned to 1 of the 4 conditions at each EMA measurement time point. In condition 1, the intervention was triggered when one of the items measuring distress (ie, negative affect, stress, loneliness, or rumination) was ≥5, which is the fixed cutoff value (on a 7-point Likert scale). We used a disjunctive decision rule, issuing an intervention when any 1 of the 4 distress indicators reached the cutoff of ≥5, to ensure that each distinct manifestation of distress, as captured by these variables, could independently trigger the intervention. In condition 2, the intervention was triggered when 1 of the items measuring distress surpassed a personalized upper control limit (UCL) of an SCC [37,42]. The calculation of SCCs is described in the subsequent subsection (Calculation of SCCs). In condition 3, the intervention was triggered when participants responded with a “yes” to the question “whether it would help them right now to talk with someone about their worries or negative feelings?” In condition 4, no intervention was triggered. The intervention was also not triggered when it was already triggered twice or more on the same day. All decision rules (fixed cutoff, SPC, and support need) were evaluated continuously (ie, in real time) during the EMA response on the participant’s device. If any rule was satisfied at the end of the EMA questions, the JITAI was delivered immediately.

#### Calculation of SCCs

We computed univariate SCCs for each distress indicator (negative affect, stress, loneliness, and rumination) as outlined by Schat et al [37]. Our implementation followed a classical 2‐phase SPC design. During the baseline phase (days 1-7), we estimated natural variability of each distress variable by computing the moving range per person (i) for each pair of successive observations at time points (j):



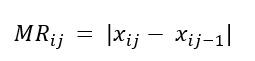



Next, we calculated the average moving range per person and divided this number by the constant (*d*_2_=1.128; defined by Montgomery [66]) to obtain the person-specific moving range–based estimate of the SD, denoted as 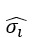 [67]. Unlike the conventional sample SD, this computation was based on changes between subsequent observations and thus represented a dynamic SD measure. The personalized UCL was then defined as follows:



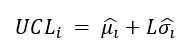



where 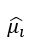 is the phase 1 mean, and the multiplier (L=2) corresponds to an approximate 1‐sided type I error rate of 2.28% per observation [66].

The intervention phase began on day 4, once each participant had completed at least 7 baseline EMA assessments (all met this criterion on day 4). From that point onward, each new EMA distress score was compared against the participant’s UCL. The JITAI was triggered if the score exceeded the UCL and the participant had received fewer than 2 interventions that day. UCLs remained constant after the “learning” phase ended (after day 7), consistent with standard SPC practice [37,66]. Consequently, the intervention time points were personalized as the computation of the UCL thresholds took into account the person-specific mean and the dynamic estimate of the SD.

Given the EMA intensity and ethical concerns around delaying support, we opted for a short baseline phase, in which interventions were available after 3 days and the SPC algorithm “learned” from the previous observations up to the first 7 days. Extending the baseline further may have improved the estimation of individual variability but would likely have reduced adherence in a sample of psychotherapy-seeking individuals. Unfortunately, there are currently no established standards for JITAI baseline durations in mental health, as this research is still in early stages [68,69].

### Data Analysis

To examine the feasibility and proximal outcomes of the social support JITAI, data were analyzed in 3 main steps. First, descriptives on feasibility and proximal outcomes were described based on grand means (across all participants), person-specific means (mean values per person), between-person SDs (SDs computed based on the person-specific means), and mean within-person SDs (mean of the SDs computed per person). Furthermore, compliance and careless responding were assessed. Compliance was defined as the proportion of completed EMA surveys out of the total scheduled surveys (126 surveys per participant across 21 days), with ≥70% of participants completing ≥70% of surveys considered sufficient. Data quality was evaluated by calculating the percentage of careless responses to an attention-check item, with high data quality defined as having <5% of responses being careless.

For aim 2, we tested whether the outcomes of appropriate timing, helpfulness, and behavior adoption differed across the 3 JITAI triggering strategies (fixed cutoff, SPC, and support need). We chose these variables because they were measured on the EMA level (appropriate timing and behavior adoption) or daily level (helpfulness). Differences were analyzed using Welch 2-sample 2-tailed *t* tests, comparing pairs of conditions, with Cohen *d* effect sizes to quantify the magnitude of differences. Analyses for helpfulness were restricted to days where only 1 intervention was triggered, as it was measured only in the evening.

Finally, for aim 3, the intervention effects on subsequent social support seeking and changes in momentary distress (negative affect, stress, loneliness, and rumination) were evaluated. Changes in distress variables were computed by subtracting the values from T+1 from the values of T. Pairwise *t* tests were conducted to compare JITAI-triggered time points to the control condition (ie, omitted JITAIs). JITAI-triggered time points included reports from conditions 1, 2, and 3, where the intervention was delivered. In contrast, the control condition (condition 4) was where no intervention was triggered, despite participants meeting at least 1 of the predefined triggering criteria (conditions 1, 2, or 3). In other words, a JITAI was considered as part of the control condition when participants (1) reported distress score more than 5 on at least 1 distress item (condition 1), (2) exceeded their personalized SPC cutoff (condition 2), or (3) indicated a need for social support (condition 3), but no JITAI was delivered because the time point was in condition 4. Cohen *d* was calculated to determine the effect size. Given the exploratory nature of this feasibility study, no formal hypothesis testing was conducted. All tests were 2-tailed with the type I error rate set to α<.05.

## Results

### Overview

**Figure 1:**
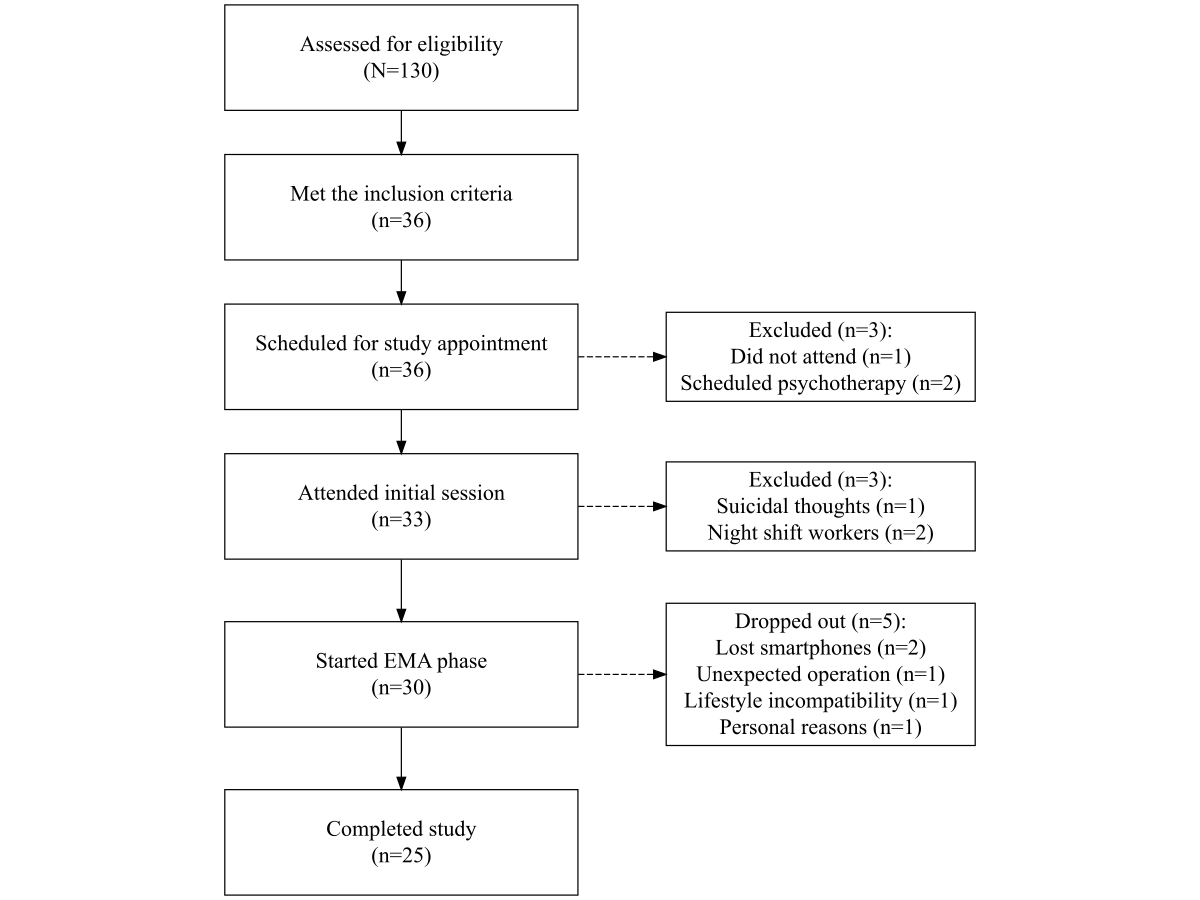
Study flowchart. EMA: ecological momentary assessment.

The flowchart of this feasibility study is presented in Figure 1. Of the 130 participants who were assessed for eligibility, 36 (27.7%) individuals met the inclusion criteria and were scheduled for a study appointment. Of these, 1 (3%) did not attend, and 2 (6%) scheduled a psychotherapy intake appointment within 2 weeks and were thus excluded from the study. During the initial session, 1 (3%) participant had to be excluded due to expressing suicidal thoughts, and 2 (6%) were excluded due to working night shifts. Over the 3-week EMA phase, 3 (8%) participants dropped out, with reasons including lost smartphones (n=2, 6%) and an unexpected operation (n=1, 3%). A total of 2 (6%) participants decided not to continue the study due to study-related factors such as lifestyle incompatibility (n=1, 3%; the person who left the study by choice indicated that the EMA content and schedule were not adapted to her lifestyle and that they found the items too impersonal) and other personal reasons (n=1, 3%). No participants were excluded based on the second interview or the minimally required EMA completion rate of 50%. Ultimately, 25 (69%) participants were included in the feasibility study.

In total, participants completed 2689 EMA reports, with an average of 107.56 (SD 20.46) reports per participant. Evening assessments yielded 232 valid responses overall, corresponding to a mean of 9.28 (SD 4.98) reports per participant. Finally, weekly reports resulted in 75 entries, with all 25 (100%) of the 25 participants completing all 3 weekly reports. The intervention was triggered 377 times in total, corresponding to n (14%) of all 2689 collected EMA time points—221 (58.6% of intervention time points) times by the fixed cutoff condition, 110 (29.2%) times by the SPC condition, and 46 (12.2%) times by the support need condition. In total, 76 JITAIs were not triggered because there were already 2 JITAIs triggered on a given day. This was the case for 15 (60%) of the 25 individuals on 55 (10%) different intervention days. The mean trigger count per person was 15.08 (SD 8.62; range 2-34). Figure S1 in Multimedia Appendix 1 shows the overlap between potential trigger moments by condition, indicating a strong overlap between potential fixed cutoff time points with SPC and support need time points. Figure S2 in Multimedia Appendix 1 exemplifies the loneliness trajectories with possible trigger time points for the fixed cutoff and the SPC condition.

Preintervention BDI-II scores ranged from 9 to 30 (mean 19.12, SD 5.87). The 25 participants fell into the following depression categories [57,58]: minimal (n=3, 12%; scores 9-13), mild (n=12, 48%; scores 14-19), moderate (n=7, 28%; scores 20-28), and severe (n=3, 12%; scores 29-63). Hence, most participants (n=19, 76%) reported either mild or moderate depressive symptoms. Postintervention scores ranged from 1 to 27 (mean 14.48, SD 8.17).

### Aim 1: Feasibility, Proximal Outcomes, and Data Quality

**Figure 2:**
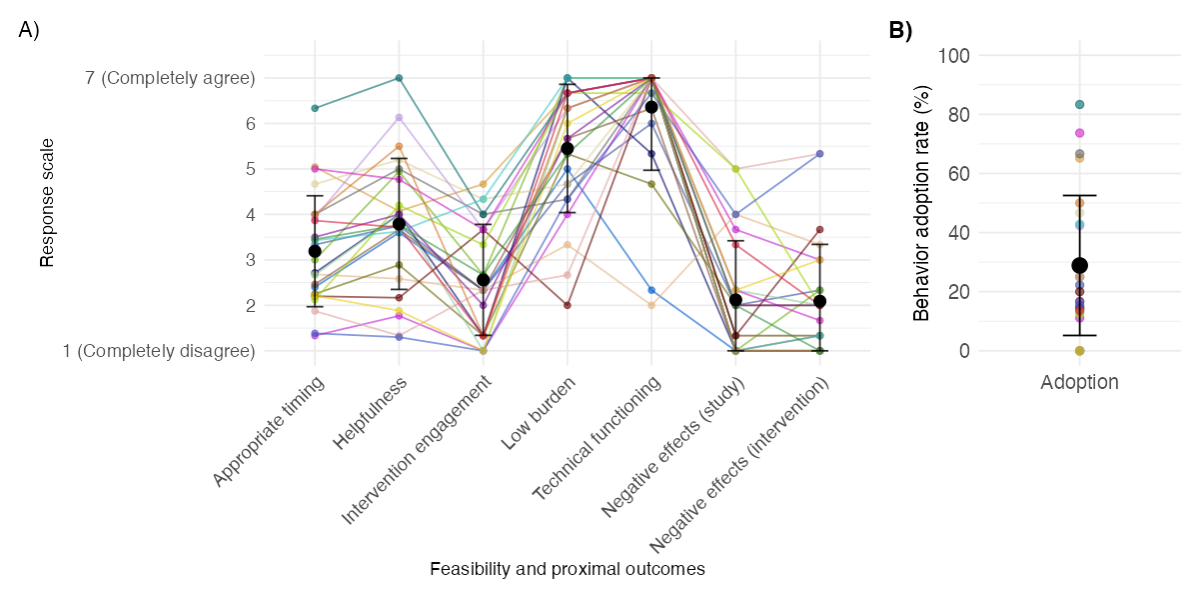
Feasibility and proximal outcome variables with person-level mean data points of (A) Likert-scale responses and (B) binary responses. In total, 25 colored data points show person-specific mean values. The technical functioning item was inversely formulated and recoded to present positive values.

The first aim of the study was to examine various feasibility and proximal outcomes, specifically how participants perceived the following: (1) appropriate timing, (2) helpfulness, (3) adoption, (4) acceptability, (5) burden, (6) technical functioning, and (7) negative effects of the social support JITAI. Figure 2 presents the grand mean and between-person SDs of the items across all participants and time points as well as the person-specific means indicated by colored data points and lines (refer to Table S2 in Multimedia Appendix 1 for the means, between-person SDs, and within-person SDs). The appropriate timing and helpfulness were rated moderately high with means of 3.19 (between: SD 1.22, within: SD 1.22) and 3.79 (between: SD 1.44, within: SD 1.00), respectively (Figure 2A). Participants reported having adopted the suggested intervention behavior to seek social support in 29% on average (between: SD 23.68) of the situations when asked at the next EMA time point (Figure 2B). If they indicated no behavior adoption, we asked for the reasons why they did not seek social support (results of a qualitative content analysis of these reports are presented in Table S5 in Multimedia Appendix 1). Intervention engagement rated on a weekly level was relatively low, with a mean of 2.56 (between: SD 1.22, within: SD 1.22). Low burden was rated with a mean of 5.45 (between: SD 1.41, within: SD 1.15) across the 3-week observation period, indicating most participants would not mind if the study went longer. The means for the low burden item on a weekly level were 2.32 (between: SD 1.95), 2.24 (between: SD 1.54), and 3.08 (between: SD 1.82), respectively. Technical functioning was rated high with a mean of 6.36 (between: SD 1.39, within: SD 0.31). Participants reported low levels of negative effects because of the study participation (mean 2.12, between: SD 1.30, within: SD 0.57) or the intervention (mean 2.09, between: SD 1.25, within: SD 0.70). However, as can be seen in Figure 2A, 2 (8%) of the 25 participants reported relatively high negative effects (above the scale midpoint of 4). In sum, in most feasibility variables, the range of person-specific means was relatively high, suggesting that our social support JITAI was perceived positively by some individuals while being perceived less positively by others.

To further evaluate the feasibility of the study, we examined the compliance rate and data quality of participants’ responses. Participants were expected to complete 126 EMA surveys over the 21-day period, with 6 surveys per day. A compliance rate was considered sufficient if ≥70% of the participants completed ≥70% of the EMA surveys. The average compliance rate across participants was 85.37% (SD 16.24%). A total of 80% (20/25) of the participants met the compliance threshold, completing at least 70.6% (89/126) of the EMA surveys.

Data quality was assessed by examining the rate of careless responses, defined as responses to an attention-check item. Out of 389 total responses, only 6 (1.5%) were classified as careless. This percentage is small, indicating that participants generally provided reliable responses.

### Aim 2: Differences Between Triggering Strategies

**Figure 3:**
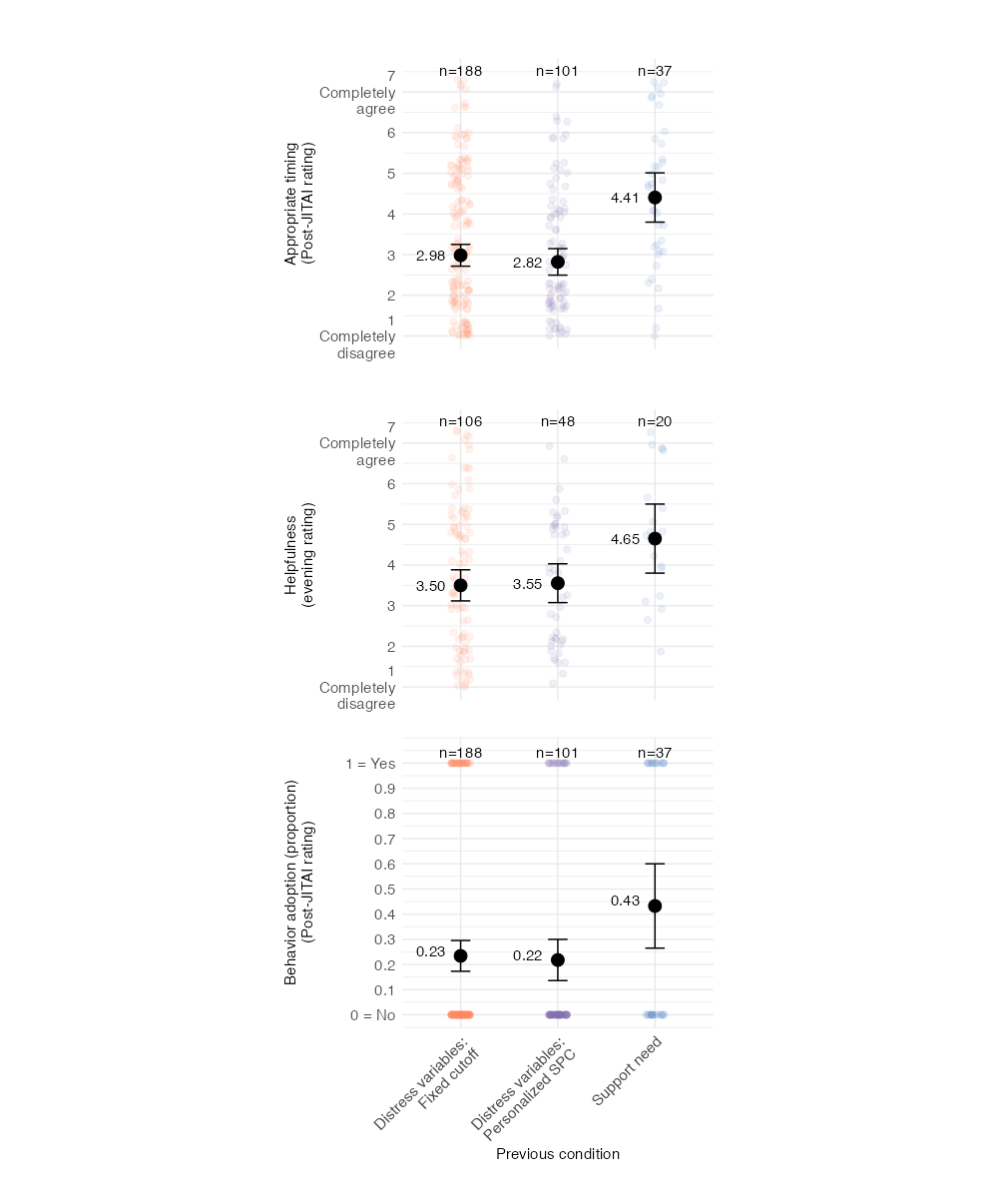
Outcomes with raw data points and means with 95% CIs by condition. Appropriate timing and behavior adoption (n=25) were assessed at the next ecological momentary assessment time point following a just-in-time adaptive intervention (JITAI). Helpfulness was rated in the evening when at least 1 JITAI was triggered. SPC: statistical process control.

We further investigated differences in the triggering conditions based on appropriate timing, helpfulness, and behavior adoption. Figure 3 shows the raw data points and grand means of these 3 variables by condition of the previously triggered JITAI. These exploratory results indicate that the appropriate timing was perceived differently depending on the triggering strategy. Specifically, appropriate timing ratings were significantly higher for the support need strategy (condition 3) compared to both the SPC strategy (condition 3: mean 4.41, SD 1.82; Cohen *d*=–1.21; *t*_59.13_=–4.64; *P*<.001) and the fixed cutoff strategy (Cohen *d*=–0.77; *t*_51.95_=–4.33; *P*<.001). Similarly, the support need strategy was associated with higher helpfulness ratings compared to both the SPC strategy (condition 3: mean 4.65, SD 1.81; condition 2: mean 3.56, SD 1.63; Cohen *d*=–0.65; *t*_32.63_=–2.33; *P*=.03) and the fixed cutoff strategy (condition 1: mean 3.49, SD 1.95; Cohen *d*=–0.60; *t*_28.29_=–2.60; *P*=.02). Regarding the adoption of support-seeking behavior when presented the JITAI, behavior adoption ratings were higher in the support need condition compared to both the SPC strategy (condition 3: mean 43%, SD 0.50%; condition 2: mean 22%, SD 0.42%; Cohen *d*=–0.49; *t*_55.01_=–2.32; *P*=.02) and the fixed cutoff strategy (condition 1: mean 23%, SD 0.42; Cohen *d*=–0.45; *t*_46.66_=–2.25; *P*=.03). These results suggested that participants perceived the timing and helpfulness of JITAI interventions as more appropriate and were more likely to adopt the behavior when interventions were triggered based on the support need item compared to the other strategies.

Because in this analysis we did not differentiate between different participants, in Figure S3 in Multimedia Appendix 1, we compare the person-level means of appropriate timing, helpfulness, and behavior adoption, indicating high between-person variability in these outcomes. In additional exploratory analyses, we examined whether timing appropriateness differed by distress indicator (negative affect, stress, loneliness, and rumination) under the fixed cutoff and SPC conditions. These comparisons are shown in Figure S4 in Multimedia Appendix 1. The figure shows that there is a tendency toward JITAIs triggered by stress and rumination to be rated higher in appropriate timing and behavior adoption than those triggered by negative affect and loneliness, independent of whether triggered by a fixed cutoff or SPC condition.

We additionally estimated multilevel models for appropriate timing, helpfulness, and behavior adoption while accounting for the nesting of repeated observations within participants, controlling for age, gender, and baseline depressive symptoms (Table S4 in Multimedia Appendix 1). Differences between support need and the distress-based conditions remained significant for appropriate timing (fixed cutoff: b=–0.82, SE 0.28; *P*=.004; SPC: b=–0.92, SE 0.31; *P*=.003) and behavior adoption (fixed cutoff: odds ratio 0.45; 95% CI 0.448-0.453 and 0.448-0.453, *P*<.001; SPC: odds ratio 0.52; 95% CI 0.516-0.522; *P*<.001) but not for helpfulness (fixed cutoff: b=–0.57, SE 0.30; *P*=.06; SPC: b=–0.52, SE 0.34; *P*=.14).

### Aim 3: Intervention Effects on Support Seeking and Changes in Distress

**Figure 4:**
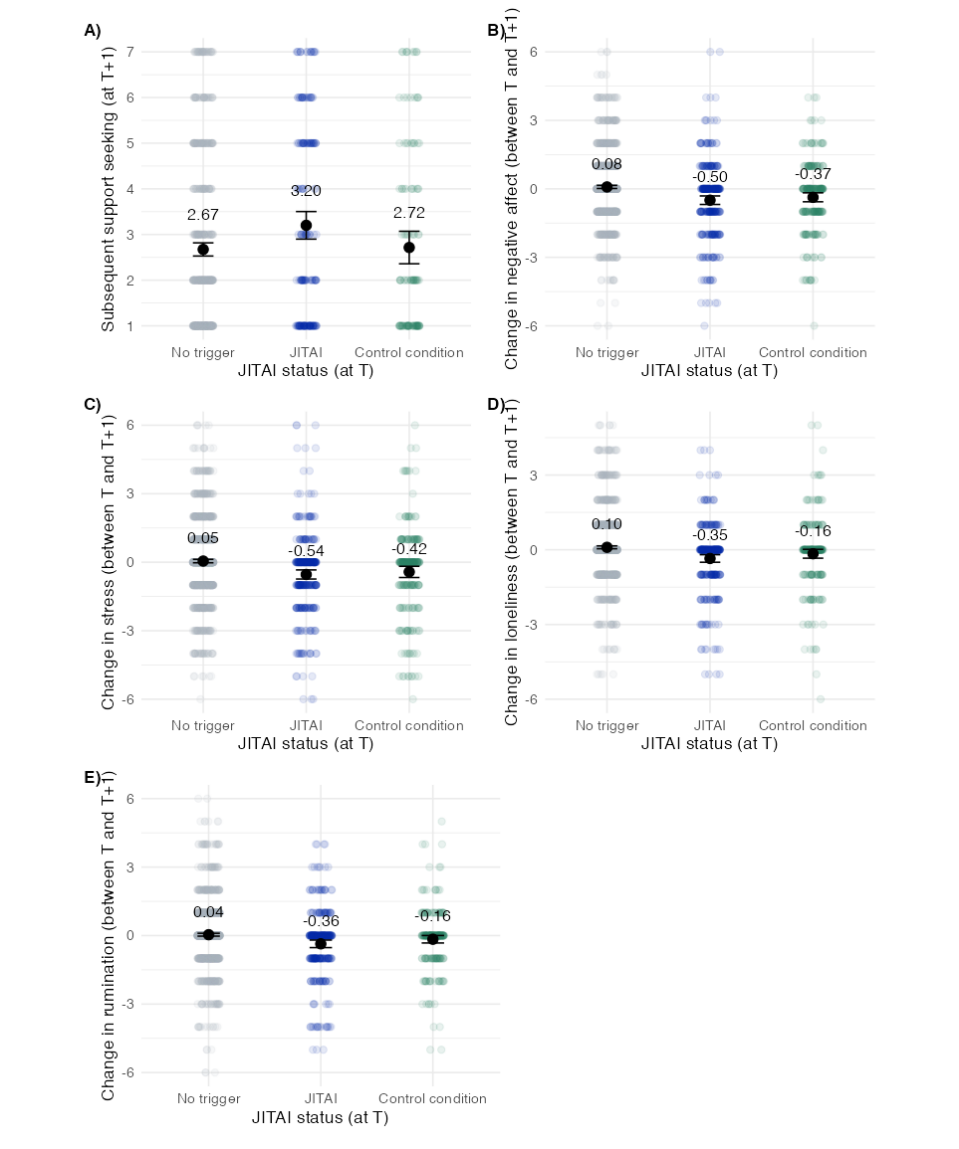
Raw data points and means with 95% CIs for sought support and changes in distress by previous triggering status. Just-in-time adaptive intervention (JITAI; n=25 individuals) triggered by either fixed cutoff, statistical process control, or support need conditions. Omitted JITAI refers to the control condition with fixed cutoff, statistical process control, or support needs condition met, but JITAI was not offered. T: previous ecological momentary assessment time point; T+1: subsequent ecological momentary assessment time point.

As this study is a feasibility study rather than a full-scale effectiveness trial, the primary aim was to assess the feasibility of implementing the JITAI and participants’ perceptions of its usability and acceptability rather than to test its impact on mental health outcomes. Nonetheless, we explored the intervention effects of the social support JITAI on the proximal outcomes of (1) sought social support and (2) changes in momentary distress measured at the subsequent EMA time point by comparing the intervention conditions (1, 2, or 3) against the control condition when the JITAI that should have been triggered by 1 of the conditions was omitted (condition 4). Figure 4 shows the raw data points and grand means of sought social support and changes in distress measures split by whether the JITAI was triggered (conditions 1, 2, or 3), not triggered, or omitted (control condition) at the previous EMA time point (ie, instances where participants met the triggering criteria but no JITAI was delivered). Figure 4A shows that support seeking at T+1 was rated higher when the JITAI was triggered (mean 3.20, SD2.13) compared to the control condition (when it was omitted) at T (mean 2.72, SD 1.99; Cohen *d*=0.24; *t*_272_=2.06; *P*=.04), indicating that the intervention had a positive effect on subsequent support seeking. Figure S5 in Multimedia Appendix 1 shows these comparisons additionally split by condition (fixed cutoff, SPC, and support need). These results indicated that subsequent support seeking was not rated higher in the fixed cutoff condition when the JITAI was triggered (JITAI: mean 3.50, SD 2.42; control: mean 2.82, SD 2.26; Cohen *d*=0.29; *t*_51.93_=1.11; *P*=.27), in the SPC condition (JITAI: mean 3.38, SD 2.24; control: mean 3.28, SD 2.18; Cohen *d*=0.05; *t*_106.99_=0.25; *P*=.81), or in the support-need condition (JITAI: mean 3.04, SD 2.01; control: mean 2.78, SD 2.02; Cohen *d*=0.13; *t*_210.61_=0.94; *P*=.35). Figures 4B-4E show the raw data points and mean changes in momentary distress variables between T and T+1 split by JITAI status (ie, not triggered, triggered, or omitted). Figures 4B-4E present the changes in negative affect, stress, loneliness, and rumination between T and T+1, comparing when the JITAI was not triggered, triggered, and omitted (ie, no JITAI condition but at least 1 criterion met for conditions 1-3). More negative change values represented larger reductions in distress. While descriptive trends suggest slightly larger reductions in these variables when the JITAI was triggered, the differences were not statistically significant in pairwise comparisons. Specifically, for negative affect (Figure 4B), changes were not significantly different between the JITAI-triggered time points (mean –0.50, SD 1.71) and the control condition (mean –0.37, SD 1.56; Cohen *d*=–0.08; *t*_546_=–0.92; *P*=.36). Similarly, for stress (Figure 4C), the change scores did not differ significantly (JITAI: mean –0.54, SD 1.83; control: mean –0.42, SD 1.94; Cohen *d*=–0.06; *t*_503.95_=–0.70; *P*=.48). Regarding loneliness (Figure 4D), participants showed a slightly larger reduction when the JITAI was triggered (mean –0.35, SD 1.39) compared to the control condition (mean –0.16; 1.41), but this difference was not statistically significant (Cohen *d*=–0.14; *t*_518.18_=–1.60; *P=*.11). Finally, for rumination, the difference in change scores approached significance, with larger reductions observed in the JITAI-triggered condition (mean –0.36, SD 1.51) compared to the control condition (mean –0.16, SD 1.30; Cohen *d*=–0.14; *t*_556.18_=–1.71; *P*=.09). Overall, the JITAI-triggered time points did not significantly differ from the control condition reducing negative affect, stress, loneliness, or rumination. Figures S6-S9 in Multimedia Appendix 1 show these comparisons additionally split by condition (ie, fixed cutoff, SPC, and support need). In addition, Figures S10-S14 in Multimedia Appendix 1 show the effects of the intervention split by whether the participants reported having sought social support because of the intervention. These figures descriptively show that the intervention effects are larger or only apparent when participants seek social support due to the JITAI as opposed to not seeking support or the intervention not being presented.

We further explored whether reductions in momentary distress differed depending on which distress variable triggered the intervention (negative affect, stress, loneliness, or rumination). Figure S16-S19 in Multimedia Appendix 1 shows these detailed comparisons. No clear pattern emerged in how conditions or triggering distress variables were associated with changes in distress variables. Furthermore, for comparisons between triggering strategies in changes in distress variables, refer to Figures S6-S9 and Table S3 in Multimedia Appendix 1.

## Discussion

### Principal Findings

This feasibility study explored the practical implementation of a social support JITAI delivered via the smartphone for psychotherapy-seeking individuals with subclinical and clinical levels of depressive symptoms. We assessed participants’ perceptions of the intervention’s feasibility and proximal outcomes while also comparing the effects of different JITAI triggering strategies on proximal outcomes, such as support-seeking behavior and changes in affective states. Exploratory analyses examining the potential intervention effects should be interpreted with caution, as the study was not designed or powered to establish efficacy.

The results suggest that the social support JITAI is practically feasible. High compliance rates were observed, with participants completing an average of 85.37% (2689/3150) of the EMA surveys. Most (20/25, 80%) participants also exceeded the preregistered compliance threshold, and the data quality was high, with a negligible proportion of careless responses (6/389, 1.5%). In addition, participants reported minimal technical issues, and burden scores indicated that completing the EMA surveys was not overly demanding. These findings are encouraging, particularly given the challenging nature of intensive data collection in real-world settings, and suggest that JITAI-based interventions can be effectively integrated into participants’ daily routines.

Despite the intervention’s technical feasibility, participants rated the overall helpfulness and appropriateness of the JITAI as moderate. Similarly, the subjective experiences of the intervention were mixed. Participants scored low on engagement in the intervention and adopted the intervention (ie, sought social support) in about 1 out of 3 times after the intervention was triggered. Barriers to seeking support, such as time constraints (ie, not having time to seek support) or perceived unavailability of support providers, may have contributed to the lower adoption rates (refer to Table S5 in Multimedia Appendix 1 for reasons provided by participants). At the same time, it is difficult to judge what the acceptable behavior adoption rates would have been for this type of intervention, given that a few more high-quality interactions may have helped to reduce depressive symptoms [70-72]. Another factor that might influence adoption rates may lie in the assumptions about what good intervention time points are, which is something that we addressed by randomizing different intervention-triggering conditions.

When zooming in on differences between triggering conditions, it seems that the JITAIs triggered based on participants’ explicit self-reported need for support (“support need” condition) were rated as more appropriate, helpful, and effective in encouraging behavior adoption than those triggered based on predefined distress thresholds (“fixed cutoff” condition) or personalized control limits (“SPC” condition), with an average Cohen *d* effect size of –0.69 (range –0.45 to –1.21) and moderately high agreement scores. These findings align with the notion that individuals are more likely to engage with an intervention when it aligns with their subjective experiences and immediate needs rather than relying solely on inferred distress [46,47]. However, it is worth noting that the support need strategy was triggered far less frequently than the other strategies, likely capturing rarer but more meaningful moments for participants. This lower frequency may contribute to the higher feasibility and adoption ratings observed in this condition, as the intervention aligned more closely with participants’ perceived relevance and immediate needs. Individuals may be more willing to seek support when they explicitly feel that they need it rather than merely experiencing distress. Feeling distressed alone may not necessarily prompt the desire or readiness to seek social support [39,73]. Particularly, not all distress is necessarily conducive to seeking support. For example, interpersonal distress may make individuals hesitant to reach out, especially if the source of distress is a conflict within their social network. By contrast, the thresholds used for the fixed cutoff and SPC conditions may have been set too low, leading to frequent interventions that participants did not perceive as necessary. This discrepancy is evident from the substantially lower frequency of reported support needs compared to the number of triggers in these conditions.

In examining the initial intervention effects on the proximal outcomes of support-seeking behavior and changes in distress variables (negative affect, stress, loneliness, and rumination), we found that receiving the social support JITAI was associated with greater self-reported subsequent support-seeking behavior, specifically, in the fixed cutoff condition but not the SPC or support-need condition. Here, support-seeking was significantly higher when the JITAI was triggered compared to the control condition. This finding suggests that the fixed-cutoff strategy may effectively identify moments of distress that prompt participants to seek social support, even if these moments do not align with participants’ explicit perceptions of need.

Furthermore, the fixed cutoff strategy may have been effective at identifying moments of distress that participants did not perceive as opportunities to seek support. Together, these findings highlight the complexity of optimizing JITAI decision rules, suggesting that different strategies may serve complementary roles in addressing both explicit and implicit needs for support. It is possible that the support need item captured a state of receptivity rather than vulnerability [20], that is, whether individuals felt ready and willing to engage with the intervention and act on its suggestions. This raises an important open question for JITAI design: Should decision rules focus solely on vulnerability or also incorporate receptivity? Notably, existing mental health JITAIs rarely account for states of receptivity [74] despite their potential relevance for intervention effectiveness. Receptive states may be particularly suitable for certain intervention types, such as social support prompts, whereas other types, such as breathing exercises, might be more beneficial when delivered during acute vulnerability. Future research should explore how different types of interventions align with and benefit from these distinct momentary states.

For changes in distress variables, JITAI-triggered time points consistently showed descriptively larger reductions in negative affect, stress, loneliness, and rumination compared to the control condition (when the JITAI was omitted). However, these differences did not reach statistical significance, likely due to the limited statistical power in this feasibility study. While the observed effect sizes (Cohen *d*=0.06-0.14) are small, even modest improvements in social support and distress reduction may be meaningful in real-world mental health interventions. Given the exploratory nature of this study, a larger trial is warranted to determine whether these effects translate into clinically significant benefits over time. These findings highlight the need for larger-scale studies to further evaluate the effectiveness of the intervention on proximal and distal outcomes while addressing potential mismatches between intervention timing and participants’ emotional states.

### Implications

The findings of this study have several implications for the design and implementation of JITAI-based interventions. First, unlike existing JITAIs that primarily focus on self-monitoring or mindfulness [74], this study uniquely targets social support activation, which is a well-documented protective factor for mental health [29,30], with a social support–specific JITAI. Second, our results underscore the importance of incorporating participants’ subjective needs into intervention decision rules. Triggering strategies that rely on participants’ self-reported support needs may be more effective and acceptable than approaches based on emotion dynamics. In addition, their effectiveness may also depend on participants’ receptiveness (ie, whether they have time) and contextual factors, such as the availability of a supportive social network or the perceived accessibility of potential support providers (as indicated by additional qualitative analysis). As evidenced by the higher ratings in the support need condition, integrating participants’ support needs into intervention decision rules could significantly enhance engagement and effectiveness. At the same time, these results underscore the need for JITAI decision rules that accurately capture participants’ subjective needs while avoiding unnecessary triggering. Overly frequent interventions, as observed in the fixed cutoff and SPC conditions, may reduce perceived appropriateness and helpfulness, potentially leading to disengagement [20]. Third, the lower-than-expected rate of behavior adoption highlights a potential gap between the intervention’s suggestions and participants’ readiness or ability to act on them. One reason might lie in the availability of time perceived by the participant and their support resources, as indicated by their reasons for not adopting the intervention (refer to Multimedia Appendix 1 for a qualitative analysis). Future iterations of the JITAI could incorporate receptivity, both the respondent’s receptivity to seeking support and the potential availability of support providers. This could include assessing whether the respondent has time to act on the support-seeking impulse and identifying which support providers are potentially available currently or later.

### Limitations

This study has several limitations. First, the study’s short duration and exploratory focus on proximal outcomes, such as behavior adoption and distress reduction, preclude conclusions about the intervention’s long-term impact on mental health outcomes. Future research should assess whether these short-term effects translate into sustained improvements in depressive symptoms. Second, the intervention focused exclusively on emotional social support, which may not address the broader range of support needs (eg, instrumental or informational support). We chose this because of the key role of emotional social support in reducing distress symptoms [75]. However, individuals with depressive symptoms may experience difficulties in accurately perceiving or using available social support due to cognitive biases, which could limit the intervention’s effectiveness [76,77]. In addition, social support may not always be skillful and thus inadvertently exacerbate distress [46,78]. Expanding the intervention’s scope to include other types of support may be beneficial.

The third limitation concerns our “support-need” trigger. Unlike distress-based triggers, which represent states of vulnerability (eg, elevated negative affect), asking participants whether they need support may not only assess vulnerability but also participants’ receptivity to this type of intervention [20]. In other words, a “yes” response could reflect readiness or willingness to engage with social support prompts while, at the same time, reflect being vulnerable. Thus, we capture 2 related but distinct constructs, namely support-need vulnerability and intervention-specific receptivity, and our item for assessing support need does not allow us to fully disentangle them. Future studies should incorporate measures that separately assess vulnerability and receptivity to refine optimal decision rules for delivering support.

Fourth, while the support-need item was intentionally brief to minimize participant burden, we acknowledge its limitation, as it remains unclear which types of distress most strongly motivate a desire for social support. However, our data suggest that participants were able to identify meaningful moments of support need. Interventions triggered by this item were rated as most appropriate and were most associated with behavioral adoption. Nonetheless, future studies should explore multi-item measures or adaptive questioning to better capture the nuances of support needs in daily life contexts.

The fifth limitation concerns the computation of SCCs as SPC triggers. Our use of the L value of 2 provided the sensitivity of the SPC trigger but may also capture milder fluctuations that are not clinically meaningful. Although other SCC applications use an L value of 3 (with α=.27%) [37], in our case, an L value of 3 would have generated only 106 intervention triggers, about 40% fewer than the 169 triggers obtained with an L value of 2. To ensure sufficient opportunities for intervention in this feasibility study, we chose the more sensitive L value of 2 as the threshold. Future studies should compare multiple L values (eg, L=2 vs L=3) and explore growing or moving-window approaches for phase 1 baseline selection to optimize the balance between sensitivity and specificity in detecting personally meaningful change.

The sixth limitation was that the sample was predominantly consisting of women, which may limit the generalizability of findings to more gender-diverse populations. Gender differences in social support preferences and help-seeking behavior [79,80] may affect intervention engagement, feasibility perceptions, and effectiveness.

The seventh limitation of this study is that the qualitative interviews conducted before the intervention and the EMA questions may have influenced participants’ awareness and expectations regarding social support. Moreover, personal contact with researchers during these qualitative interviews may have enhanced engagement and contributed to the low study attrition observed.

Finally, individuals with depressive symptoms often experience sadness, anhedonia, rumination, social withdrawal, and reduced social functioning [76,77], all of which can impair motivation and capacity to seek social support [39,81]. This challenge was a core motivation for developing our social support JITAI. However, important differences may exist among individuals with varying severity of depressive symptoms. Consequently, our findings may not generalize to those with more severe or chronic depressive symptoms, and future studies should assess differential patterns of engagement with this social support JITAI across clinical subgroups.

### Future Research

An important finding is the heterogeneity among individuals in the reported feasibility and the effects of the JITAI, a pattern that is also observed in other JITAIs and digital mental health interventions [68,82]. This indicates that the JITAI may be feasible and effective for some but not for others. Consequently, examining individual differences in the feasibility and effectiveness of the intervention (eg, baseline levels of distress and social support availability) could help identify (1) subgroups of participants who are most likely to benefit from specific JITAIs and (2) factors that might inform the personalization of future JITAIs [68,82,83]. Examining individual differences concerning the effectiveness of such interventions is crucial to optimize care [84].

Future iterations of the JITAI could also integrate components of CBT or psychoeducation to incorporate a broader range of coping strategies. For instance, making the intervention adaptive to whether a supportive person is available and whether support is perceived to be helpful for the participants or suggesting CBT elements, if no support providers are available, could be used to further tailor the intervention. Moreover, artificial forms of social support through generative artificial intelligence chatbots [85,86] would also be worth exploring, as would more specific populations, such as individuals rehabilitating after (inpatient) psychiatric care. Both of these extensions were mentioned by participants in the qualitative interviews as ideas for future development.

### Conclusions

This study supports the feasibility of implementing a social support JITAI in real-world settings and provides preliminary results about its proximal effects. By addressing the critical gap in leveraging social support for mental health, this intervention represents a step toward personalized and scalable digital mental health interventions. In summary, the findings suggest that the JITAI is practically feasible, supporting its integration into participants’ daily lives. The intervention appears to effectively encourage individuals to seek support when they recognize a personal need for it, with participants rating the JITAI as moderately helpful and appropriate under such conditions. However, prompting support-seeking behavior may be less effective, appropriate, and helpful when individuals experience elevated levels of negative affect, stress, loneliness, or rumination. In addition, the results indicate that the JITAI does not significantly reduce distress indicators in daily life, suggesting that further refinement of the social support JITAI may be needed to address its effectiveness. By providing support in real time during critical moments, this approach aligns with the call for innovative solutions to bridge the gap between demand and accessibility in mental health care [1,5]. While this JITAI is not a substitute for professional care, it offers a low-threshold, accessible, and feasible intervention that could complement existing mental health support by fostering proactive social connectedness, provided its effectiveness is supported in future studies.

## Appendix

Multimedia Appendix 1 Detailed description of the intervention, full results from the qualitative content analysis on reasons for not seeking social support after receiving the just-in-time adaptive intervention, as well as additional descriptive tables, figures, and supplementary multilevel analyses.

## Abbreviations

BDI-II: Beck Depression Inventory-II
CBT: cognitive behavioral therapy
EMA: ecological momentary assessment
JITAI: just-in-time adaptive intervention
OSF: Open Science Framework
SCC: Shewhart control chart
SPC: statistical process control
T: previous ecological momentary assessment time point
T+1: subsequent ecological momentary assessment time point
UCL: upper control limit

## Data Availability

The datasets generated or analyzed during this study are available in the Open Science Framework repository [40].
