# Supplementary material for: A Social Support Just-in-Time Adaptive Intervention for Individuals With Depressive Symptoms: Feasibility Study With a Microrandomized Trial Design
Source: JMIR Ment Health. 2025 Aug 26;12:e74103. doi: 10.2196/74103 (PMC12421209; doi:10.2196/74103)
Supplement: Multimedia Appendix 1 [file mental_v12i1e74103_app1.pdf]

Supplementary Materials

A Social Support Just-In-Time Adaptive Intervention for Individuals with Depressive Symptoms: A Feasibility Study with Micro-Randomized Trial Design

Timon Elmer[1], Markus Wolf[1], Evelien Snippe[2], & Urte Scholz[1]

[1] University of Zurich, Department of Psychology, Zurich, Switzerland

[2]University of Groningen, University Medical Center Groningen (UMCG), Department of Psychiatry, Groningen, The Netherlands

Correspondence concerning this article should be addressed to Timon Elmer,

University of Zurich, Applied Social and Health Psychology, Binzmühlestrasse 14/ Box 14, 8050 Zurich, Switzerland. Email: timon.elmer@uzh.ch

ORCIDs

Timon Elmer https://orcid.org/0000-0003-4354-4457

Markus Wolf https://orcid.org/0000-0002-5660-6824

Evelien Snippe https://orcid.org/0000-0002-3003-7475

Urte Scholz https://orcid.org/0000-0003-0184-5921

Table S1

Frequency and Percentages of Demographic Variables

Characteristic	Category	*n*	%
Gender	Female	22	88
	Male	3	12
	Other (inter, non-binary, questioning, trans)	0	0
Ethnicity	White	21	84
	Black	0	0
	Asian	0	0
	Hispanic	0	0
	Mixed	1	4
	Other	0	0
	Don't know	1	4
	None	2	8
Education	No	0	0

Attended compulsory school but did not complete it	1	4
Compulsory school	1	4
1-year training/bridging offer	0	0
Technical secondary school/Diploma secondary school	0	0
Apprenticeship / vocational school / commercial school	5	20
High school diploma/teacher training seminar	4	16
Vocational/technical baccalaureate	1	4
Professional examination with federal professional certificate/higher professional examination with federal diploma/master diploma	3	12
Higher technical college (HF)	1	4
Bachelor: University, ETH, University of Applied Sciences, University of Education (including Diploma FH/PH)	5	20
Master: University, ETH, University of Applied Sciences, University of Teacher Education (including Licentiate, Diploma University/ETH)	4	16

	Doctorate/Habilitation	0	0
	Not specified	0	0
Relationship Status	single	15	60
	in a non-registered relationship	6	24
	married/ in a registered partnership	2	8
	divorced/ dissolved partnership	2	8
	widowed	0	0
Living Situation	Living alone	7	28
	Living with partner	8	32
	Living with friends	0	0
	Living in a shared apartment	9	36
	Living with children	3	12

Note. Categories are labeled as they were presented to participants. Percentages may not sum to exactly 100 due to rounding.

Table S2.

Grand Mean and Standard Deviation of Feasibility Outcomes

Outcome	Data Source	Item [Engl. Translation]	*Mean*	$SD^{between}$	SD^{within}
Appropriate timing	Post JITAI EMA	The last time the app made me aware that I could seek social support, it was just at the right time	3.19	1.22	1.49
Helpfulness	Evening Survey	The app's instructions for activating social support was helpful	3.79	1.44	1.00
Adoption	Post JITAI EMA	Did you ask others for support because of the app's advice?	28.87[1]	23.68	-
Intervention engagement	Weekly Survey	I have asked others for social support because of the app advice	2.56	1.22	1.22
Low Burden	Weekly Survey	I don't mind completing the questionnaires for another week	5.45	1.41	1.15

Technical functioning	Weekly Survey	I had technical problems using the m-Path app	6.36	1.39	0.31
Negative effects (study)	Weekly Survey	Answering the questionnaires of this study has worsened my mental health	2.12	1.30	0.57
Negative effects (interventio n)	Weekly Survey	Asking my social contacts for help has worsened my mental health	2.12	1.30	0.57

Note. SD = Standard Deviation. All items are rated on a 7-point Likert scale from 1 (“disagree completely”) to 7 (“agree completely”) unless indicated otherwise. [1] Percentage of “Yes” responses.

Table S3

Pairwise Comparisons of Change in Distress by Triggering Condition

Distress Variable	Comparison	M1	SD1	M2	SD2	*t*	*df*	*p*
Negative Affect	Fixed vs SPC	-0.30	1.72	-0.73	1.53	2.31	236.61	.022
Negative Affect	Fixed vs Support Need	-0.30	1.72	-1.07	1.79	2.66	63.59	.010
Negative Affect	SPC vs Support Need	-0.73	1.53	-1.07	1.79	1.10	74.30	.274
Stress	Fixed vs SPC	-0.42	1.77	-0.33	2.01	-0.40	190.07	.689
Stress	Fixed vs Support Need	-0.42	1.77	-1.00	2.25	1.63	57.18	.108
Stress	SPC vs Support Need	-0.33	2.01	-1.00	2.25	1.73	77.15	.087
Loneliness	Fixed vs SPC	-0.29	1.28	-0.49	1.57	1.14	178.49	.256

Loneliness	Fixed vs Support Need	-0.29	1.28	-0.52	1.66	0.89	56.75	.379
Loneliness	SPC vs Support Need	-0.49	1.57	-0.52	1.66	0.11	81.20	.914
Rumination	Fixed vs SPC	-0.45	1.46	-0.25	1.70	-1.03	186.19	.303
Rumination	Fixed vs Support Need	-0.45	1.46	-0.50	1.60	0.21	61.59	.838
Rumination	SPC vs Support Need	-0.25	1.70	-0.50	1.60	0.87	89.96	.387

Note. M = Mean; SD = Standard Deviation; df = degrees of freedom, numbers in column labels refer to the order of condition named in comparison column.

Table S4

Multilevel Regression Results for Aim 2 Outcomes

	Appropriate Timing (Post JITAI Rating)			Helpfulness (Evening Rating)			Adoption (Post JITAI Rating)		
Predictors	Estimates	std. Error	P	Estimates	std. Error	p	Odds Ratios	std. Error	P
Intercept	4.75	0.95	<.001	4.40	1.24	0.001	1.35	0.00	<.001
Age (centered)	-0.01	0.02	.569	-0.01	0.03	0.664	1.01	0.00	<.001
Gender [ref. female]	-0.19	0.83	.819	-0.30	1.12	0.789	0.11	0.00	<.001
Depressive symptoms at baseline	-0.04	0.05	.411	-0.01	0.07	0.897	0.97	0.00	<.001
Fixed cutoff condition [ref. support need]	-0.82	0.28	.004	-0.57	0.30	0.060	0.45	0.00	<.001
SPC condition [ref. support need]	-0.92	0.31	.003	-0.52	0.34	0.135	0.52	0.00	<.001
Random Effects									
σ2	2.26			1.35			3.29		
τ00	1.19 id			2.26 id			0.56 id		
ICC	0.35			0.63			0.14		
Observations	326			169			326		
Marginal R2 / Conditional R2	0.056 / 0.382			0.023 / 0.635			0.223 / 0.335		

Note. N = 25.

Figure S1.

Venn Diagram of Potential Trigger Moments by Condition

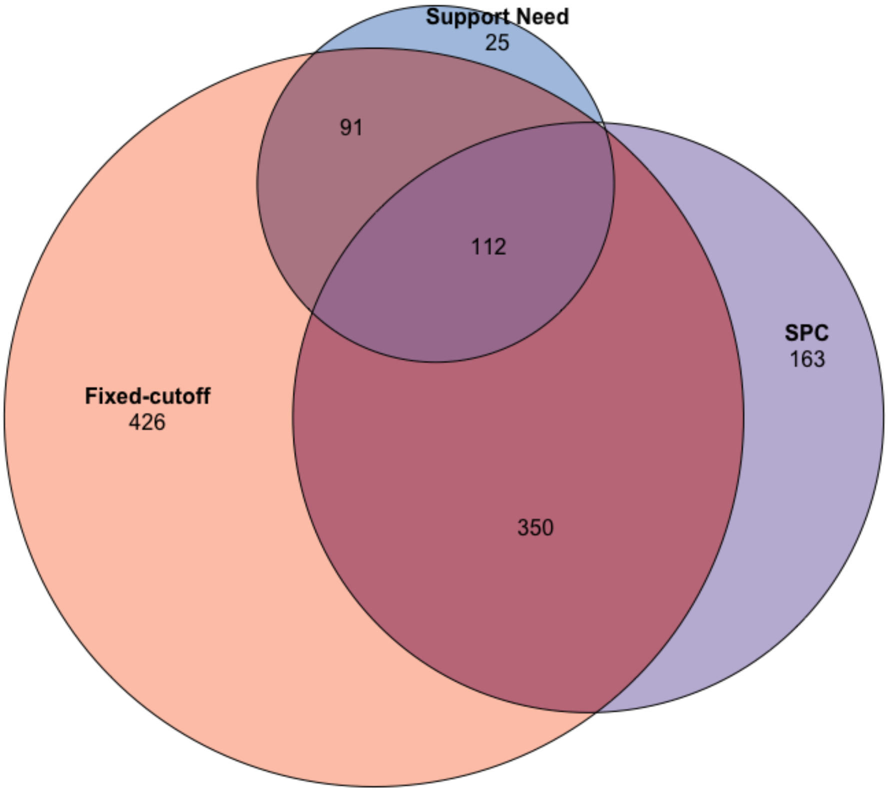

Figure S2.

Exemplary Loneliness Data of One Participant with Possible Fixed-Cutoff and SPC Timepoints

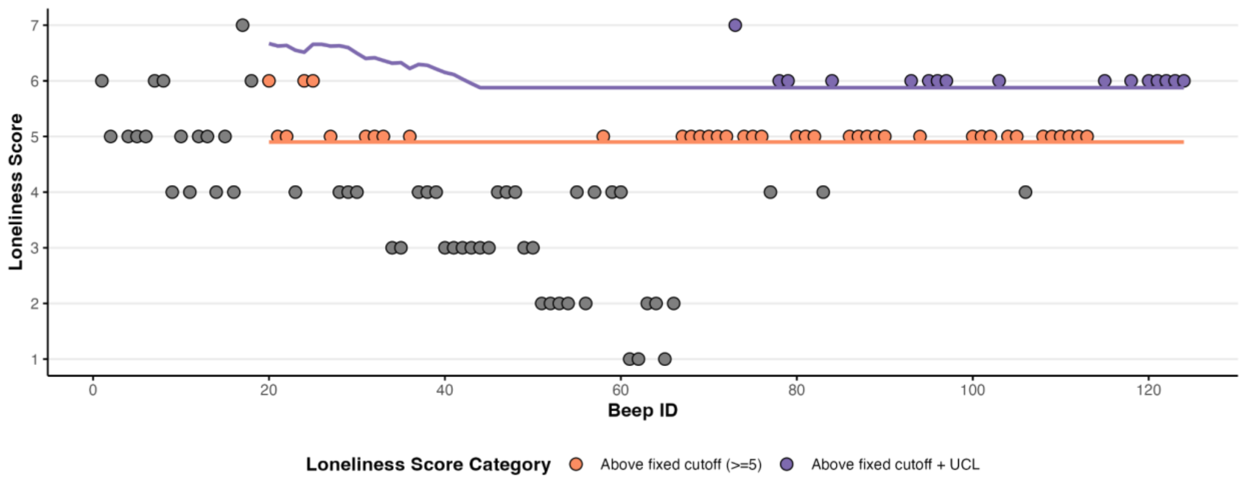

Note. UCL = Upper Control Limit of Shewhart Control Chart

Figure S3.

Outcomes by Condition with Person-Level Means

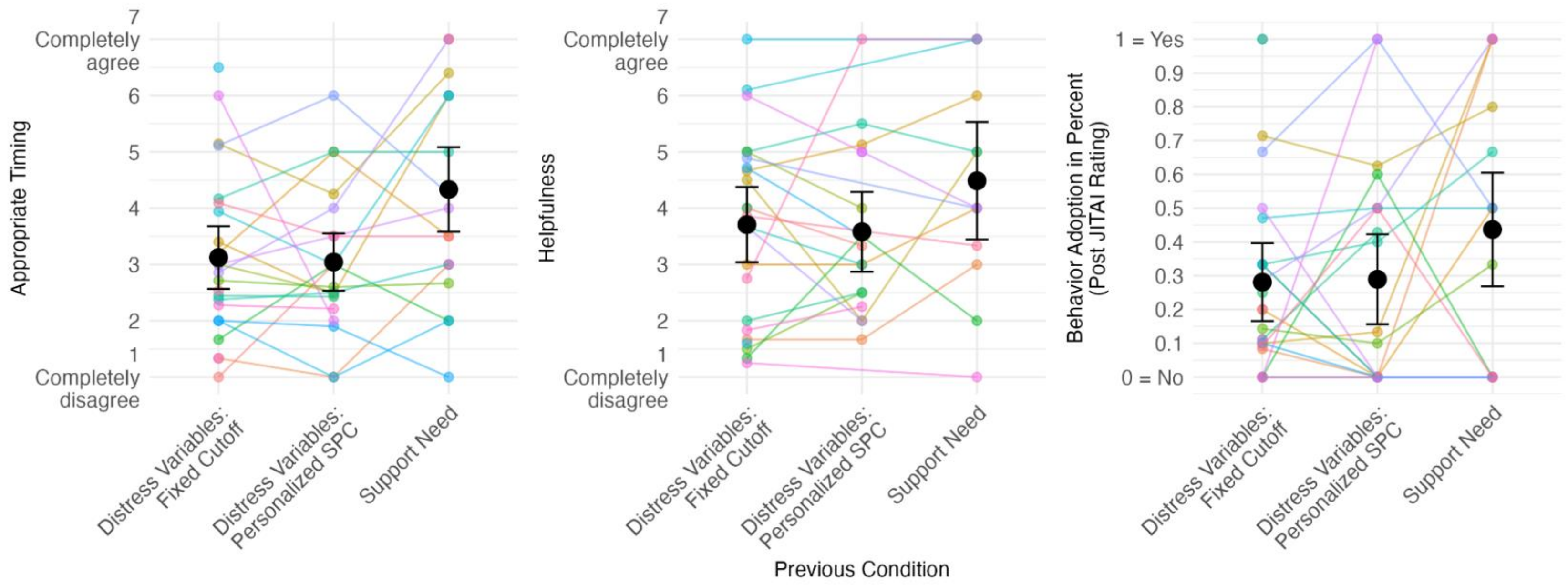

Note. Appropriate timing and behavior adoption were asked at the next EMA timepoint following a JITAI. Helpfulness was rated in the evening when at least one JITAI was triggered.

Figure S4.

Appropriate Timing and Behavior Adoption by Distress Variable Triggering the JITAI

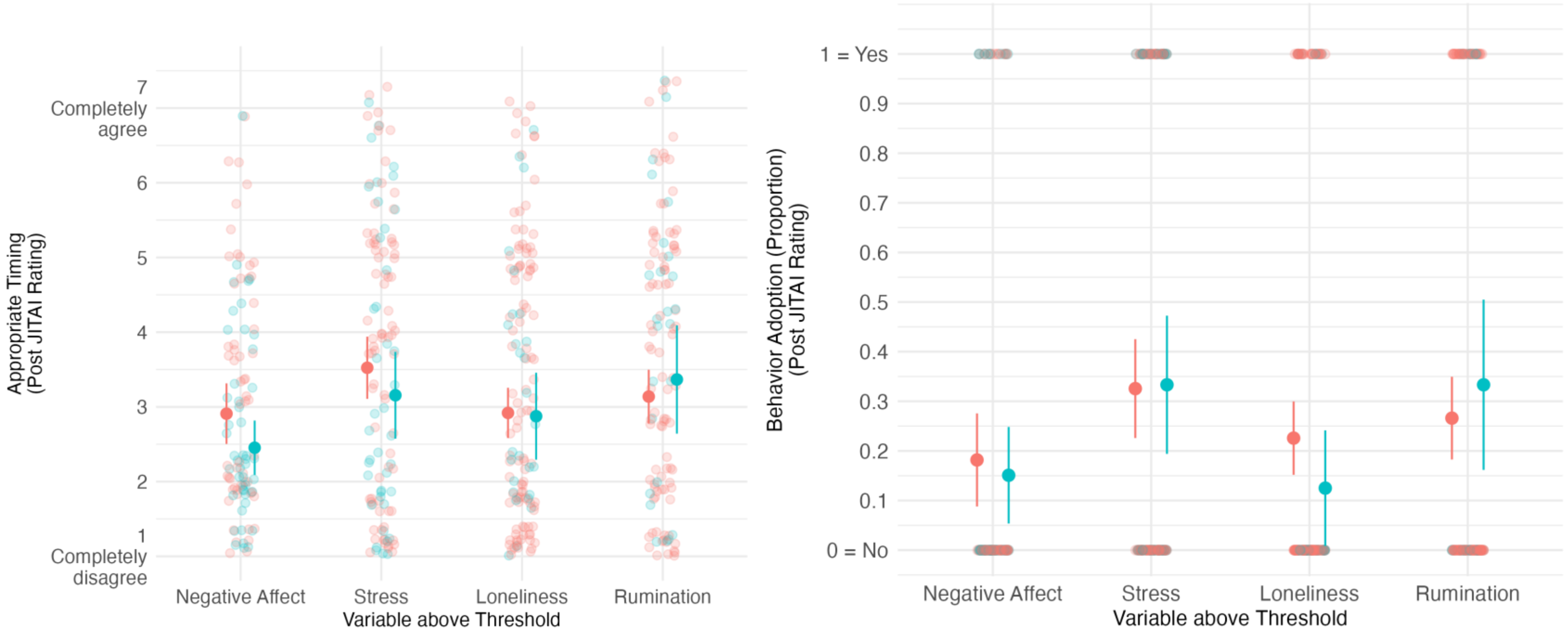

Note. Appropriate timing and behavior adoption were asked at the next EMA timepoint following a JITAI.

Figure S5.

Subsequent Support Seeking by JITAI Triggering Status Split by Condition

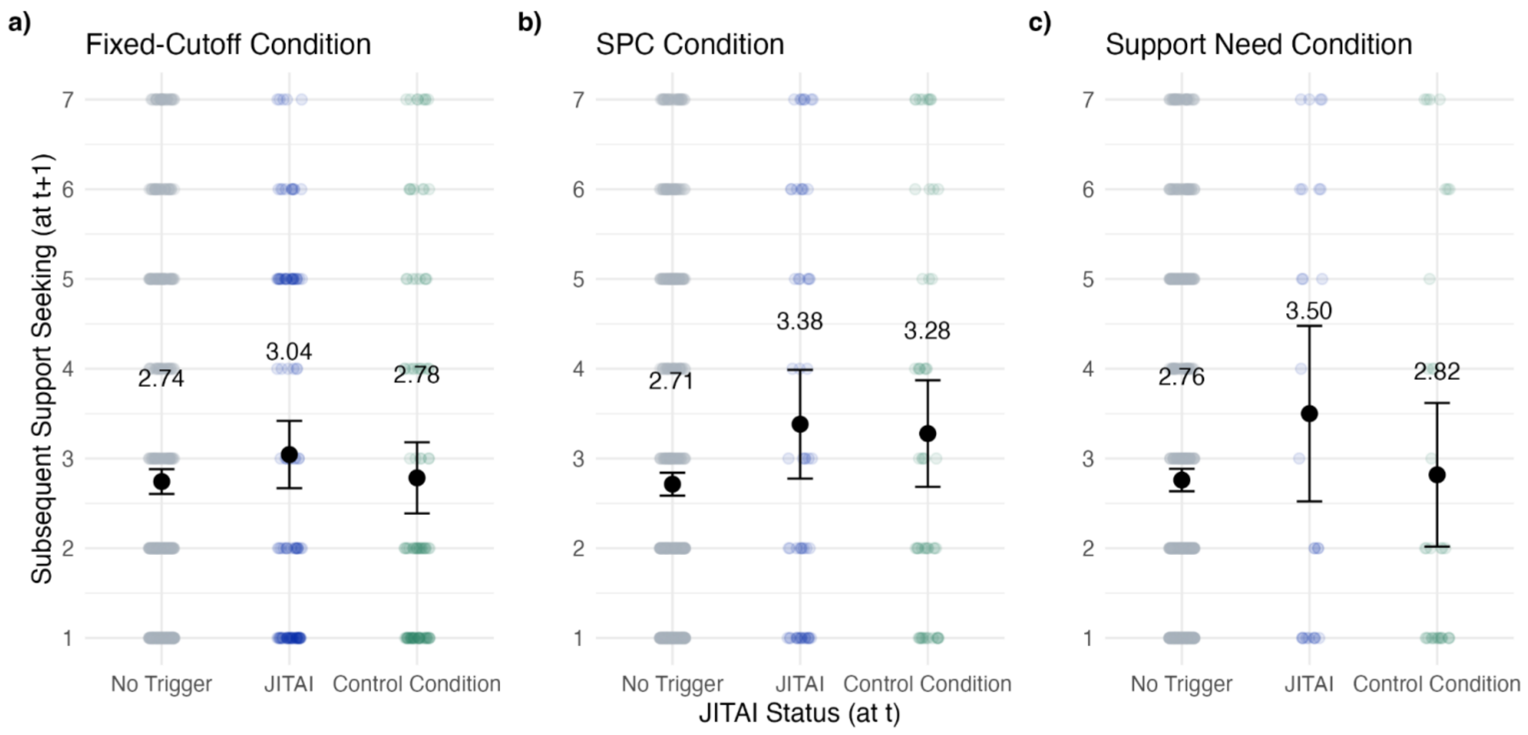

Note. N_{ind} = 25, JITAI = Just-In-Time Adaptive Intervention triggered by either fixed-cutoff, SPC, or support need conditions. ontrol condition = Fixed-cutoff, SPC, or support need condition met, but JITAI was not presented.

Figure S6.

Change in Negative Affect by JITAI Triggering Status Split by Condition

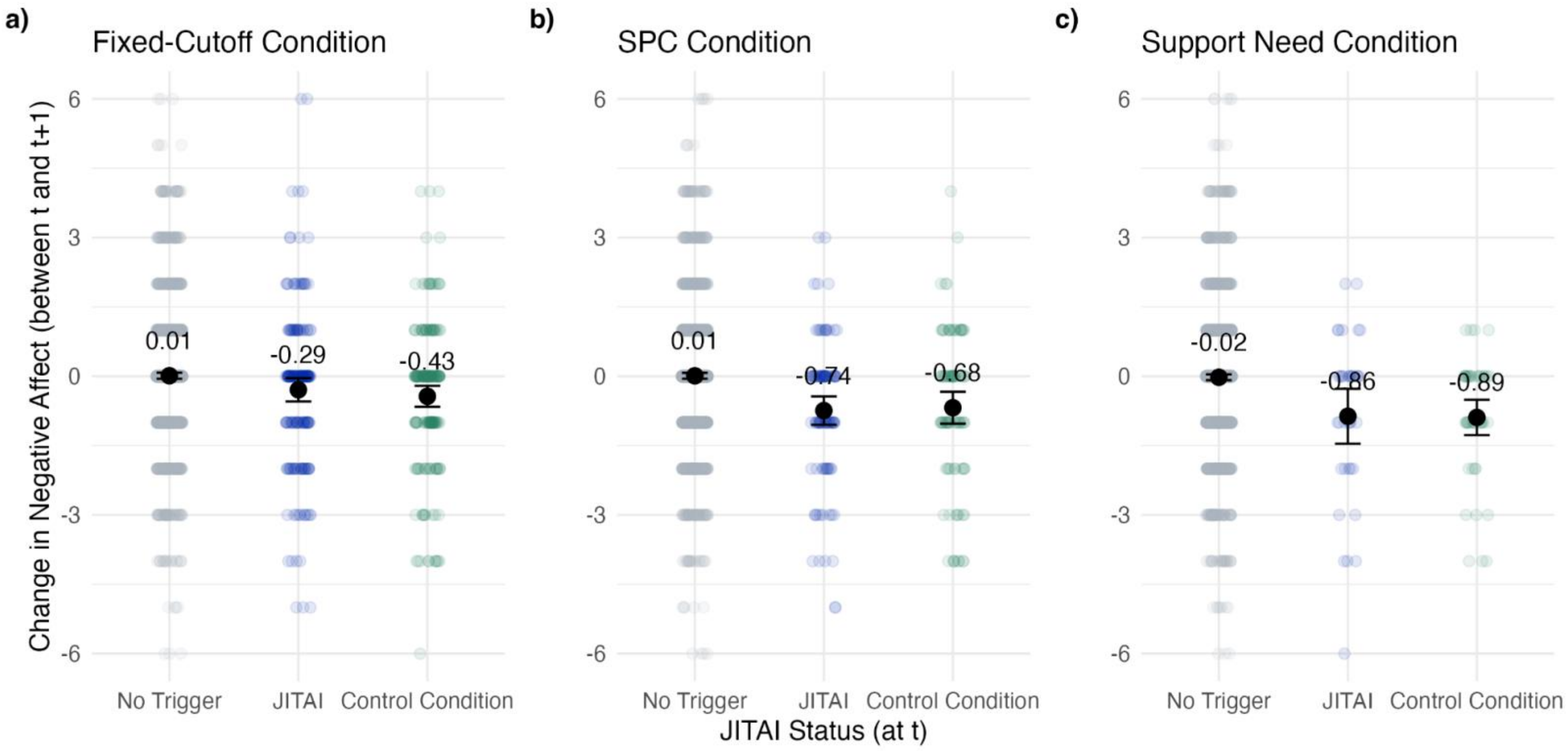

Note. N_{ind} = 25, JITAI = Just-In-Time Adaptive Intervention triggered by either fixed-cutoff, SPC, or support need conditions. Omitted JITAI = control condition with fixed-cutoff, SPC, or support need condition met, but JITAI was not presented.

Figure S7.

Change in Stress by JITAI Triggering Status Split by Condition

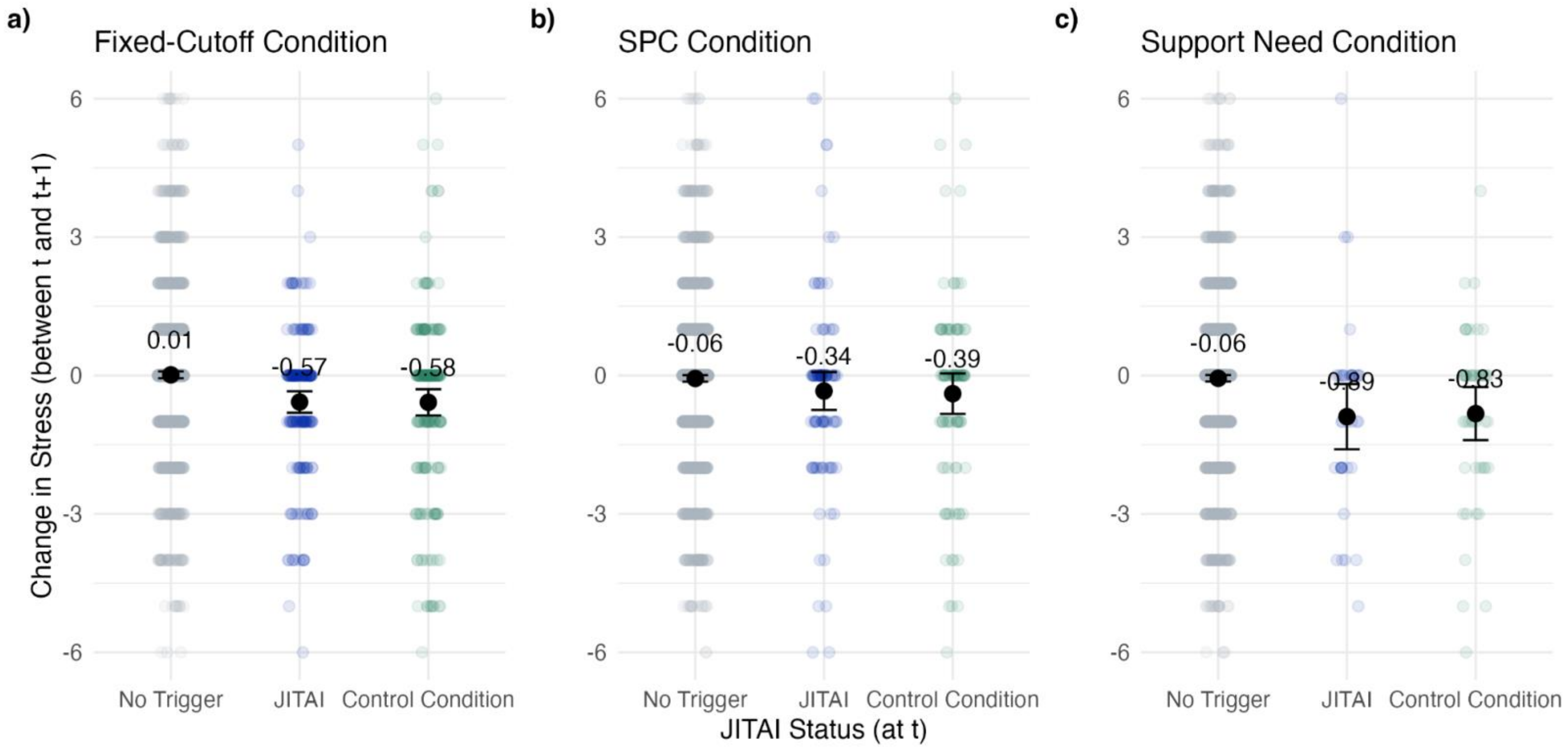

Note. N_{ind} = 25, JITAI = Just-In-Time Adaptive Intervention triggered by either fixed-cutoff, SPC, or support need conditions. Omitted JITAI = control condition with fixed-cutoff, SPC, or support need condition met, but JITAI was not presented.

Figure S8.

Change in Loneliness by JITAI Triggering Status Split by Condition

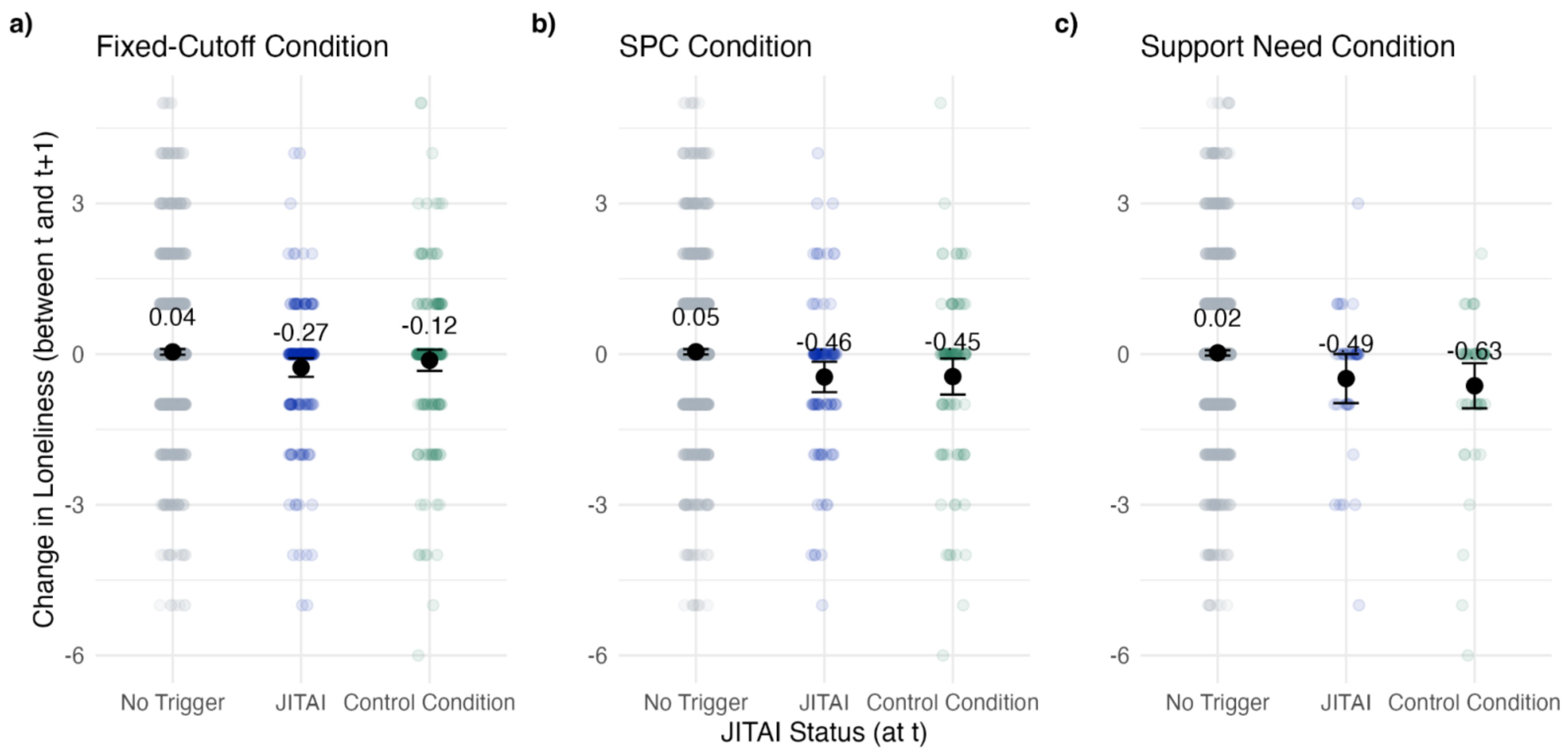

Note. N_{ind} = 25, JITAI = Just-In-Time Adaptive Intervention triggered by either fixed-cutoff, SPC, or support need conditions. Omitted JITAI = control condition with fixed-cutoff, SPC, or support need condition met, but JITAI was not presented.

Figure S9.

Change in Rumination by JITAI Triggering Status Split by Condition

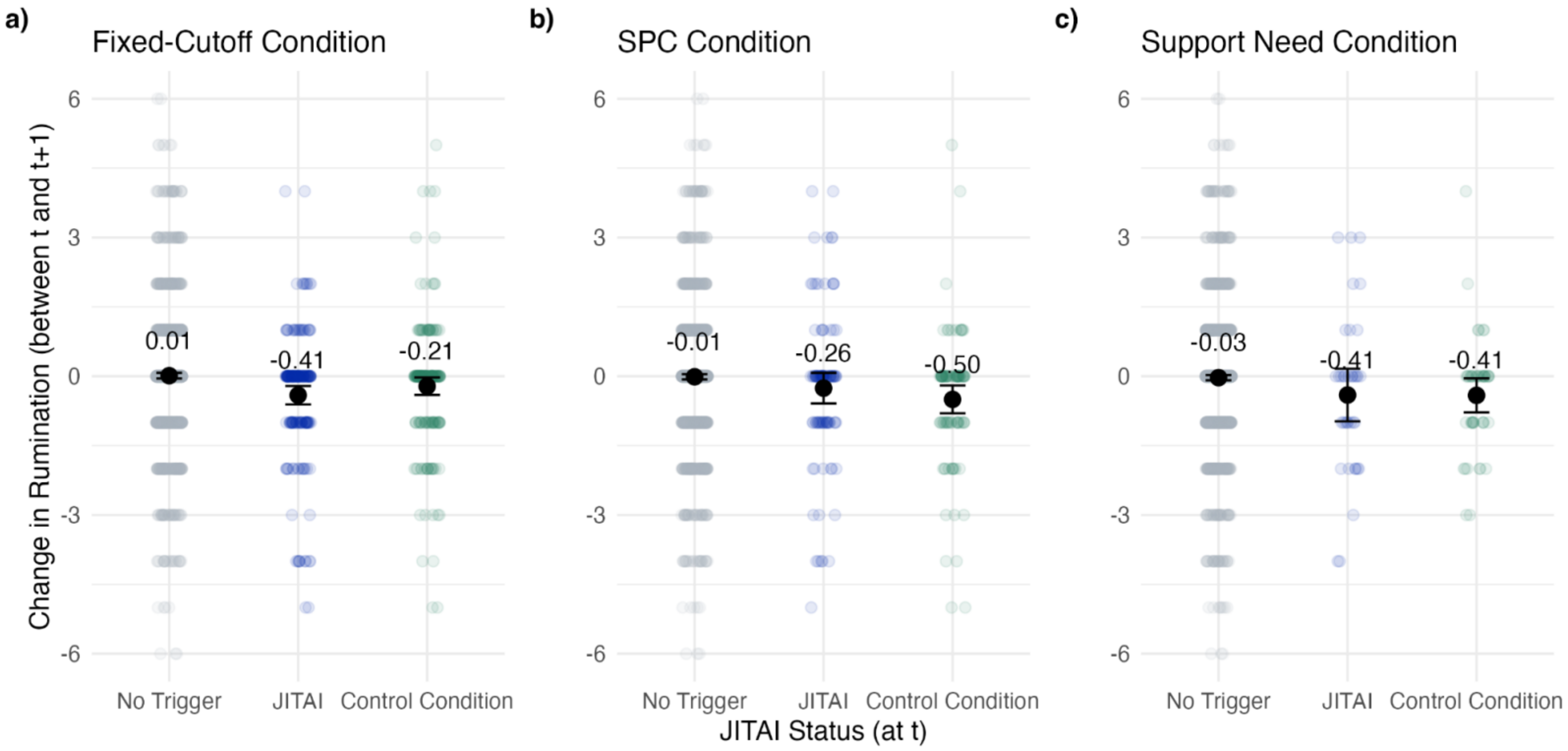

Note. N_{ind} = 25, JITAI = Just-In-Time Adaptive Intervention triggered by either fixed-cutoff, SPC, or support need conditions. Omitted JITAI = control condition with fixed-cutoff, SPC, or support need condition met, but JITAI was not presented.

Figure S10.

Subsequent Support Seeking (at t+1) by JITAI Status (at t) and Whether Intervention Behavior Was Adopted (i.e., Support Seeking)

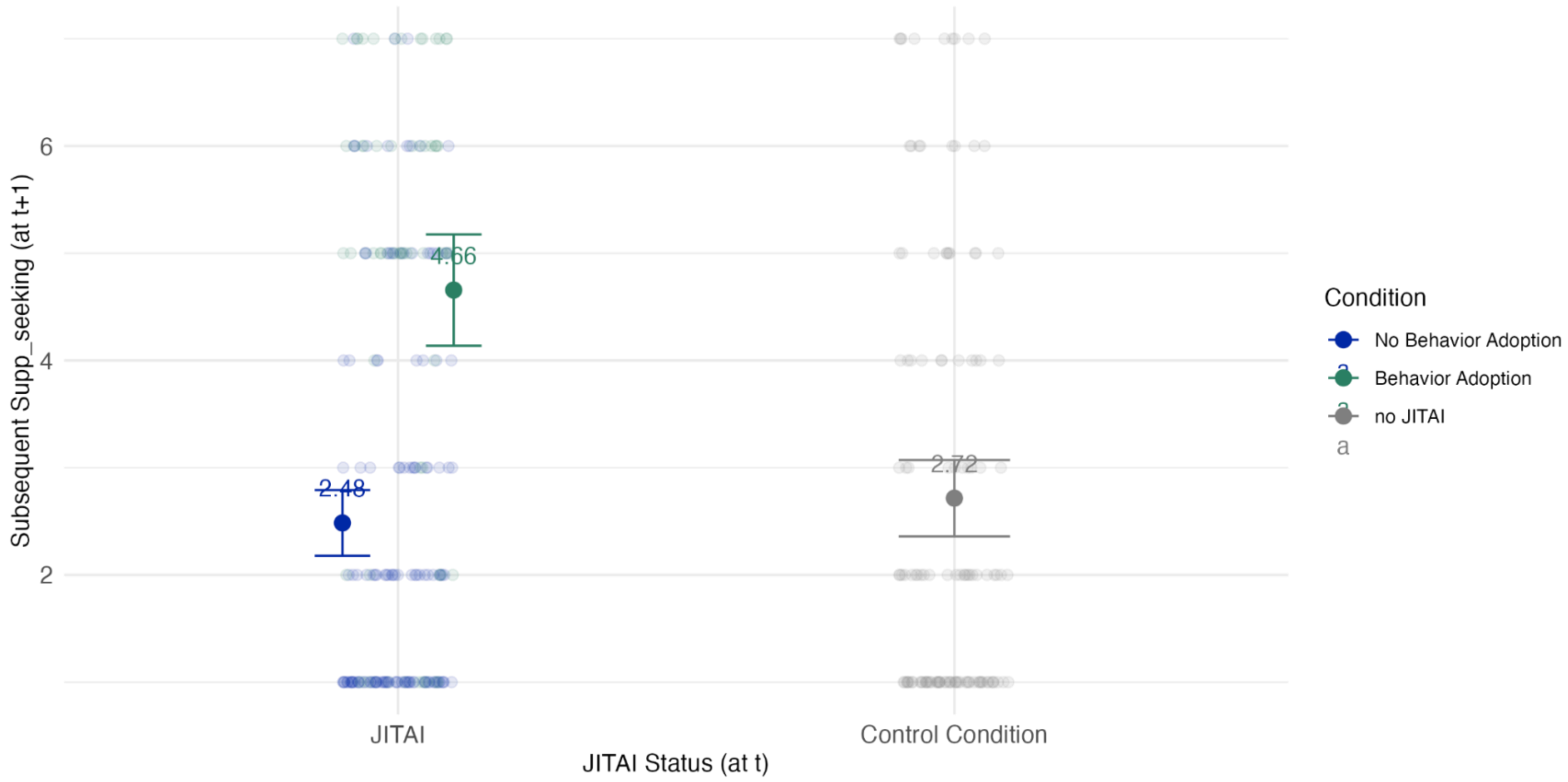

Note. N_{ind} = 25, JITAI = Just-In-Time Adaptive Intervention triggered by either fixed-cutoff, SPC, or support need conditions. Omitted JITAI = control condition with fixed-cutoff, SPC, or support need condition met, but JITAI was not presented.

Figure S11.

Change in Negative Affect (at t+1) by JITAI Status (at t) and Whether Intervention Behavior Was Adopted (i.e., Support Seeking)

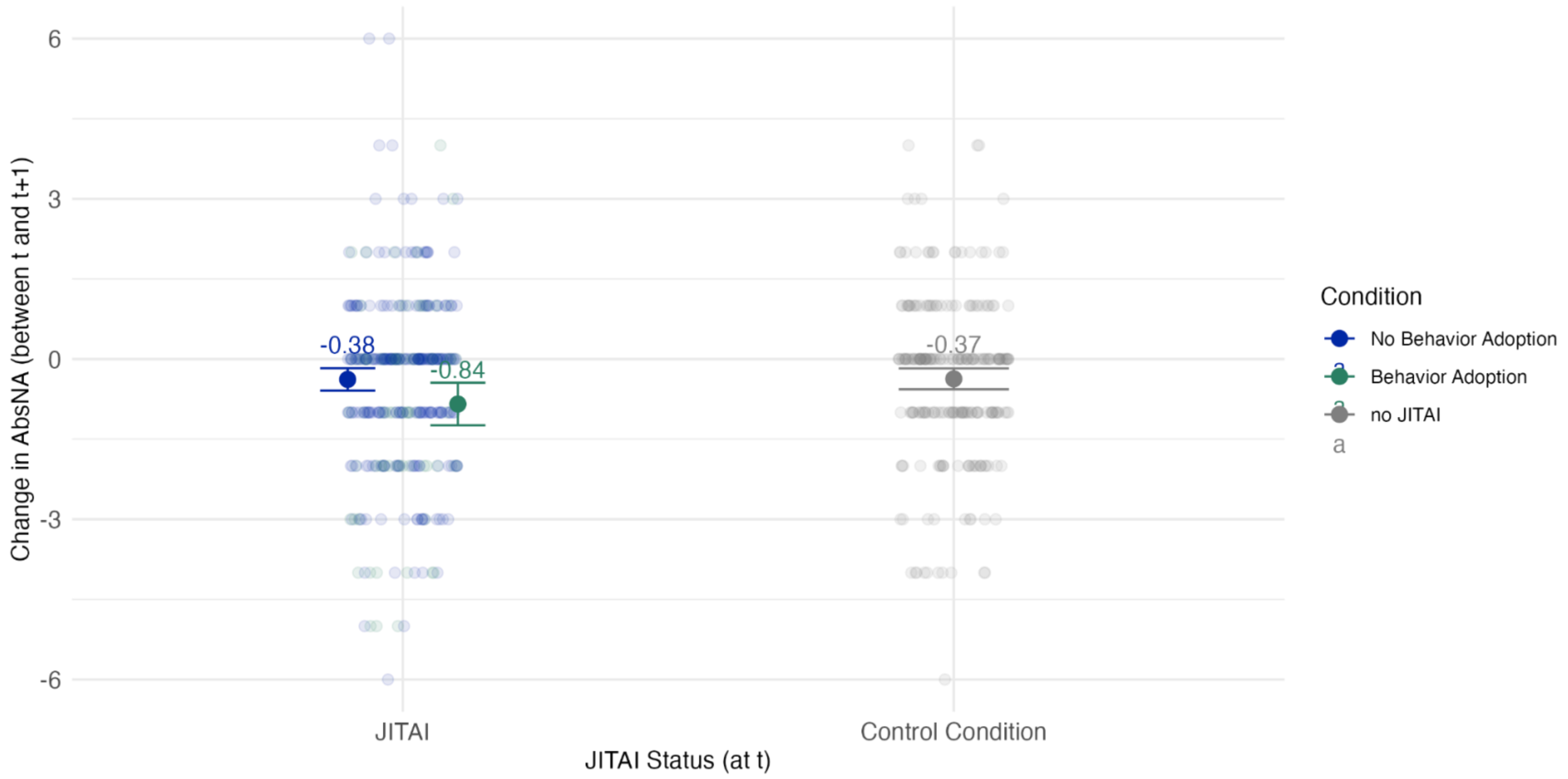

Note. N_{ind} = 25, JITAI = Just-In-Time Adaptive Intervention triggered by either fixed-cutoff, SPC, or support need conditions. Omitted JITAI = control condition with fixed-cutoff, SPC, or support need condition met, but JITAI was not presented.

Figure S12.

Change in Stress (at t+1) by JITAI Status (at t) and Whether Intervention Behavior Was Adopted (i.e., Support Seeking)

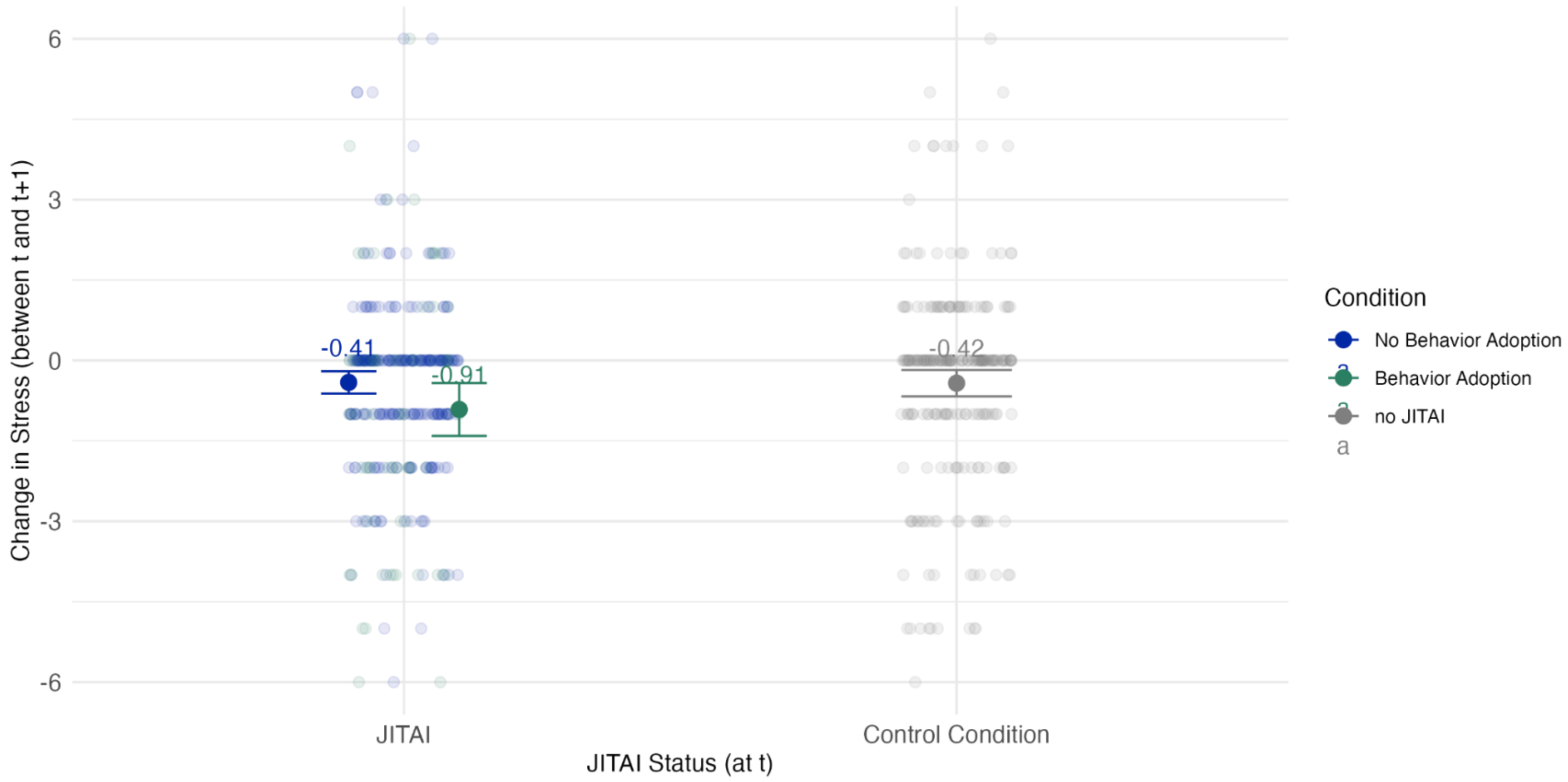

Note. N_{ind} = 25, JITAI = Just-In-Time Adaptive Intervention triggered by either fixed-cutoff, SPC, or support need conditions. Omitted JITAI = control condition with fixed-cutoff, SPC, or support need condition met, but JITAI was not presented.

Figure S13.

Change in Loneliness (at t+1) by JITAI Status (at t) and Whether Intervention Behavior Was Adopted (i.e., Support Seeking)

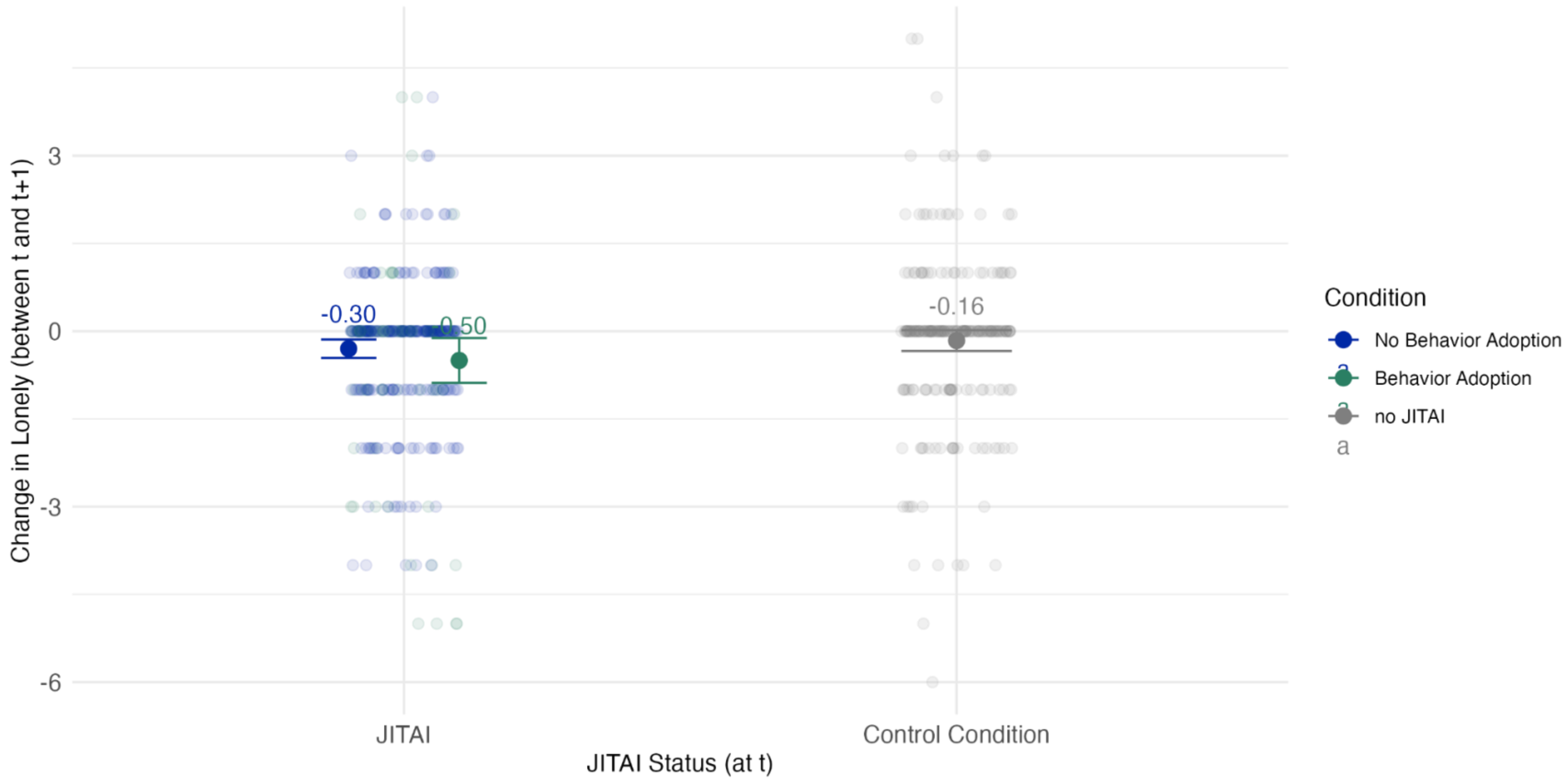

Note. N_{ind} = 25, JITAI = Just-In-Time Adaptive Intervention triggered by either fixed-cutoff, SPC, or support need conditions. Omitted JITAI = control condition with fixed-cutoff, SPC, or support need condition met, but JITAI was not presented.

Figure S14.

Change in Rumination (at t+1) by JITAI Status (at t) and Whether Intervention Behavior Was Adopted (i.e., Support Seeking)

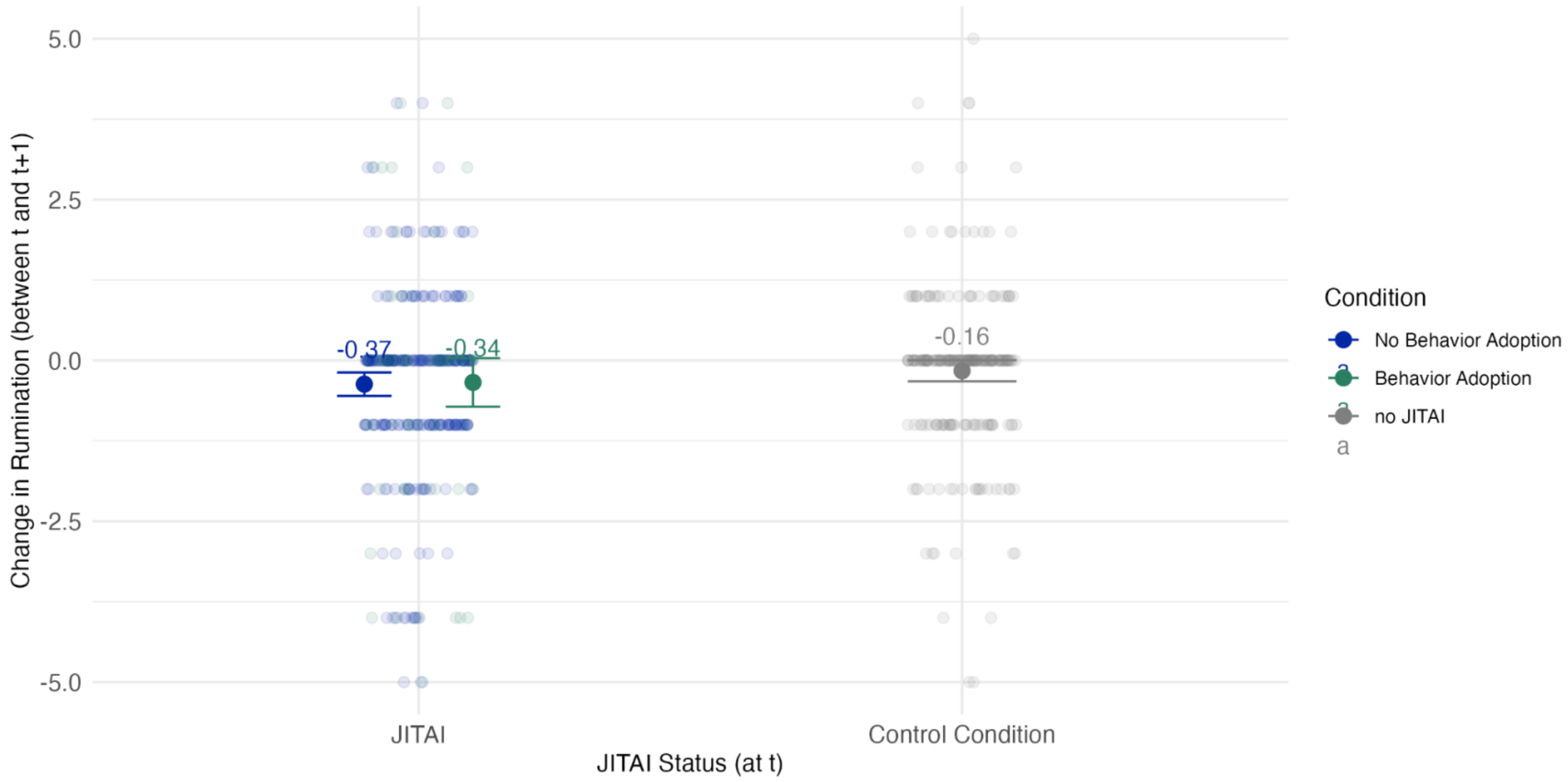

Note. N_{ind} = 25, JITAI = Just-In-Time Adaptive Intervention triggered by either fixed-cutoff, SPC, or support need conditions. Omitted JITAI = control condition with fixed-cutoff, SPC, or support need condition met, but JITAI was not presented.

Figure S15.

Support Seeking (at t+1) by Indication of Distress Variable Being Above Threshold (at t) and Condition (at t)

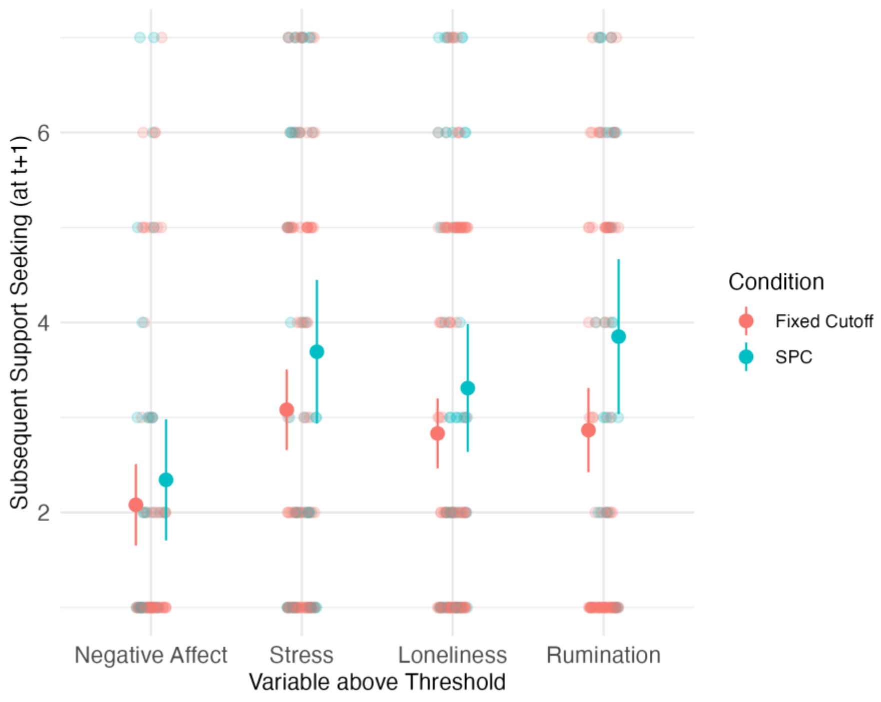

Note. Small, transparent data points are raw data points. Larger point with error bars represent the Mean and 95% Confidence Interval. Variable above Threshold at t was indicated if either the score was above 5 (fixed cutoff condition) or above the personalized UCL of the Shehwart Control Chart (SPC condition). Change on the y-axis represents the change between timepoint t and t+1.

Figure S16.

Change in Negative Affect (From t to t+1) by Indication of Distress Variable Being Above Threshold (at t) and Condition (at t)

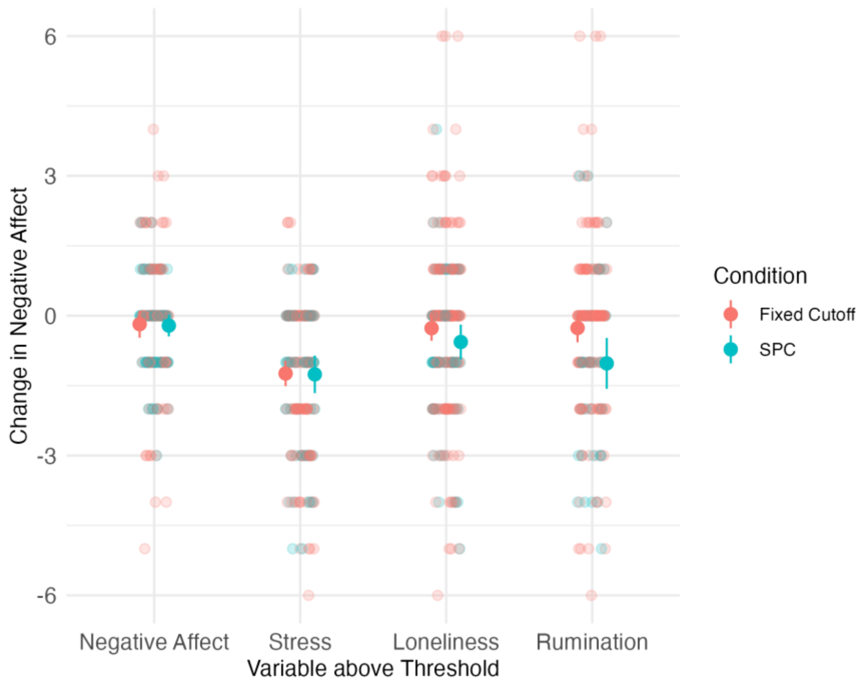

Note. Small, transparent data points are raw data points. Larger point with error bars represent the Mean and 95% Confidence Interval. Variable above Threshold at t was indicated if either the score was above 5 (fixed cutoff condition) or above the personalized UCL of the Shehwart Control Chart (SPC condition). Change on the y-axis represents the change between timepoint t and t+1.

Figure S17.

Change in Stress (From t to t+1) by Indication of Distress Variable Being Above Threshold (at t) and Condition (at t)

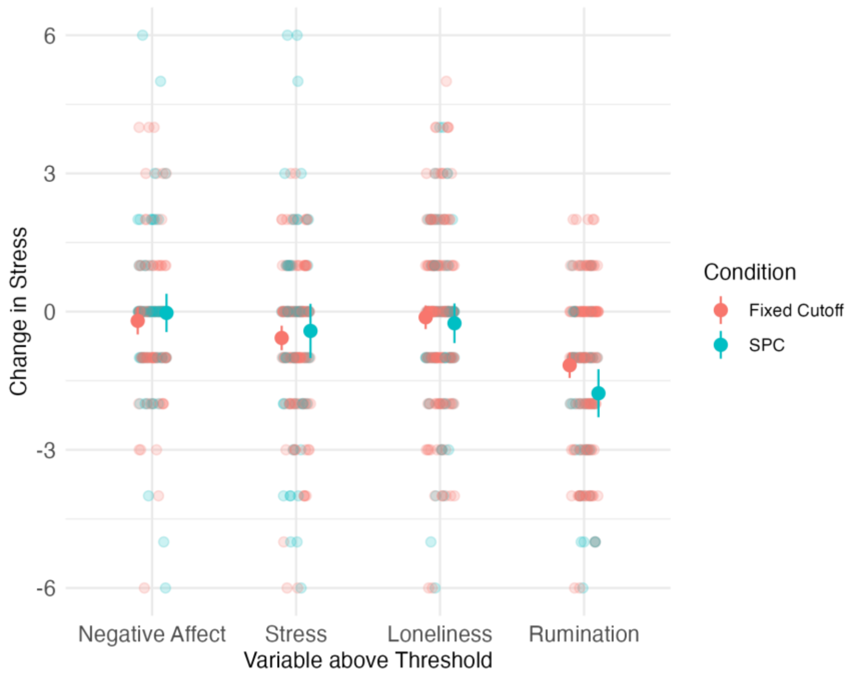

Note. Small, transparent data points are raw data points. Larger point with error bars represent the Mean and 95% Confidence Interval. Variable above Threshold at t was indicated if either the score was above 5 (fixed cutoff condition) or above the personalized UCL of the Shehwart Control Chart (SPC condition). Change on the y-axis represents the change between timepoint t and t+1.

Figure S18.

Change in Loneliness (From t to t+1) by Indication of Distress Variable Being Above Threshold (at t) and Condition (at t)

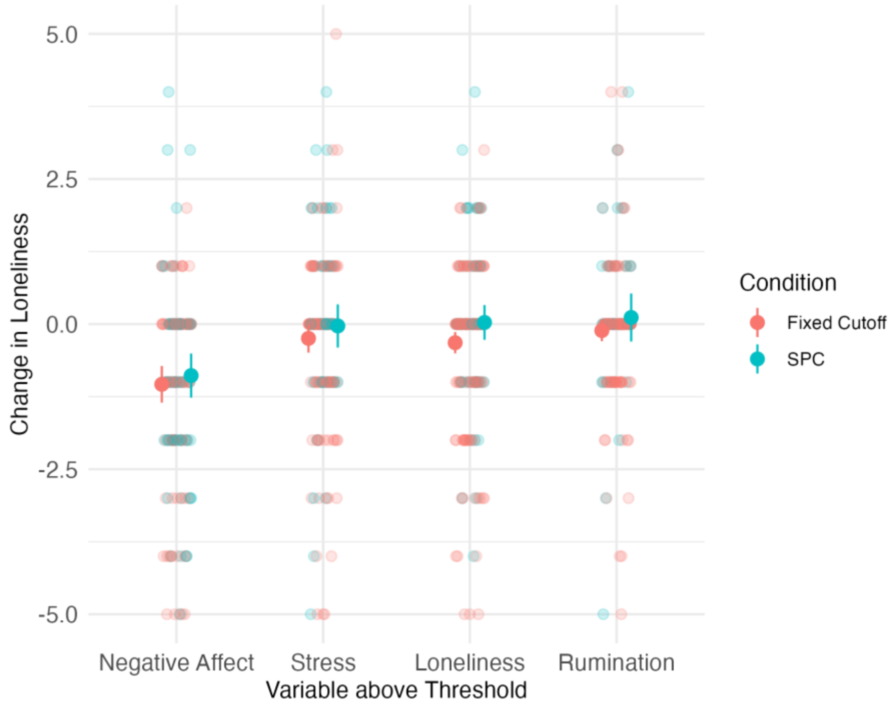

Note. Small, transparent data points are raw data points. Larger point with error bars represent the Mean and 95% Confidence Interval. Variable above Threshold at t was indicated if either the score was above 5 (fixed cutoff condition) or above the personalized UCL of the Shehwart Control Chart (SPC condition). Change on the y-axis represents the change between timepoint t and t+1.

Figure S19.

Change in Rumination (From t to t+1) by Indication of Distress Variable Being Above Threshold (at t) and Condition (at t)

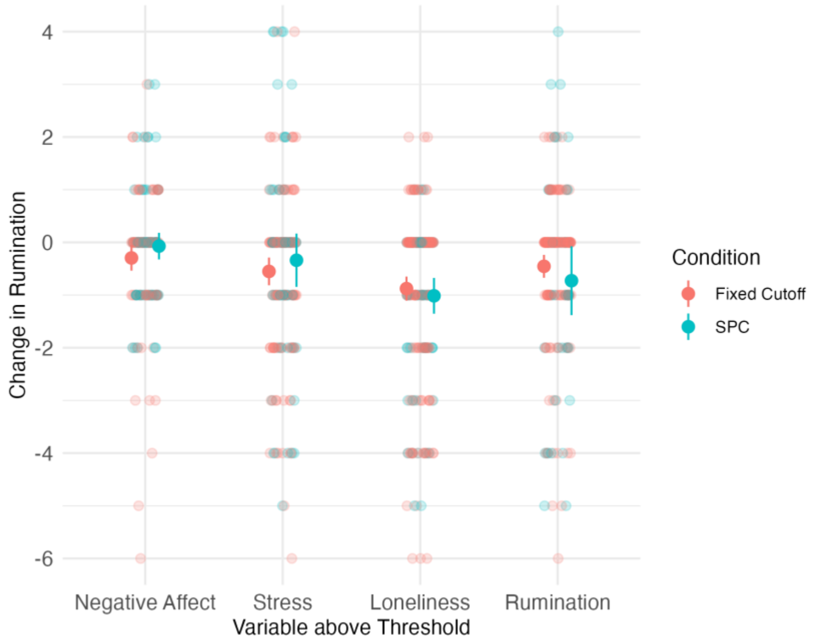

Note. Small, transparent data points are raw data points. Larger point with error bars represent the Mean and 95% Confidence Interval. Variable above Threshold at t was indicated if either the score was above 5 (fixed cutoff condition) or above the personalized UCL of the Shehwart Control Chart (SPC condition). Change on the y-axis represents the change between timepoint t and t+1.

Intervention Content (translated from German original)

German original can be found on OSF.

You have indicated that you [(Condition A + B) are not feeling well right now / (Condition C) could use help right now]. We know that in such situations it can be helpful to ask other people from your social environment for support.

What would help you to feel better at this moment? You can mention social support activities such as counseling, advice, information, listening, comforting, physical affection (e.g. hugging), practical help, doing something together, distraction or anything else you can think of.

Open Text _____

Which person from your social environment could help you with this right now?

Single-choice (self-learning): Social interaction items

You have indicated that [Person X] could best support you with [Support Type]. We encourage you to act on this suggestion and contact [Person X] now and ask him/her directly if he/she can help with [Support Type]? Asking other people for social support can be difficult. So we've put together a few pieces of advice for you.

Here is advice #[1-6] [For each intervention time, one of the advices is presented in the following order]

Advice #1: Be direct and clear about how the other person could support you.

Explanation: If you tell the other person exactly what you need, they will be better able to help you.

Concrete examples:

“ Can I tell you something briefly? It would help me if you would just listen to me for a moment.”

“I'm having problems with my work and would like to hear your opinion.”

“I need someone to help me fill out my tax return.”

Scientific background:

If you need help, just tell people directly. If you just hint or wait for them to ask you if you need help, you are more likely not to get help (Barbee & Cunningham, 1995; Pearlin & McCall, 1990).

Research has shown that it is more effective if you express your desire for help directly. When you do this, people understand what you need and are more willing to help you (Marshall et al., 2023; Rains et al., 2021; Williams & Mickelson, 2008).

If you ask for help indirectly, only make hints or give subtle clues. This can lead to people not knowing what you need or feeling unsure about how to help you (Krueger & Forest, 2020).

If you ask for help directly, say exactly what you need. This makes it easier for people to help you (Forest 2021; Krueger & Forest, 2020).

Advice #2: Talk about what is bothering you.

Explanation: Talking about your problems can make you feel better. However, excessively dwelling on problems without seeking solutions can be counterproductive and may discourage others from effectively supporting you. Instead, try to find concrete solutions with the other person.

Specific examples:

“I'm feeling very down right now and would appreciate it if you would listen to me.”

“I'm overwhelmed by all the tasks I have to deal with and feel overwhelmed as a result. Can we think together about how I can set my priorities and use my time more efficiently?”

Scientific background:

Talking negatively (e.g. complaining, whining) can put off the people who could help you. This may be because it makes them feel uncomfortable and they may think they can't help you (Forest et al., 2014; see also Wood & Forest, 2016).

Advice #3: Try to reframe the situation together with the other person

Explanation: When you talk to another person about your problems, you could specifically ask them for a different perspective on your problem. This can help you to look at your own situation in a different way.

Specific examples:

“Can I share what's bothering me, and could you help me see it from a different perspective?”

Scientific background:

When talking to others about negative feelings, in some cases it can be helpful to reframe the situation and challenge negative thoughts rather than dwelling on the negative feelings. Research suggests that reframing negative experiences can help to reduce stress and promote well-being (e.g., Brans et al., 2014; Lee et al., 2020; Pauw et al., 2018).

Reframing means changing your perspective on a situation to see it in a more positive or constructive light. This can be done by identifying and challenging negative thoughts, finding alternative ways to interpret events and focusing on the positive aspects of the situation.

Instead of focusing on the fact that you made a mistake at work, for example, you could reframe the situation by saying to yourself: "I have learned from my mistake and am now better equipped for the future."

Advice #4: Say thank you and let the other person know if the support was helpful.

Explanation: Saying thank you and giving feedback on the effectiveness of the support shows the other person that you appreciate their help.

Concrete examples:

"Thank you for listening to me. It helped me a lot."

"Your support has really helped me. I really appreciate that."

"Thank you very much for listening to me. It means a lot to me that you took the time for me."

Scientific background:

When supporters feel that they have helped, they are more likely to do so in the future (Walsh & Forest, 2021). When someone offers you support, take a moment to thank them. This simple act can increase the likelihood of further support in the future. Feeling valued and appreciated motivates people to extend their support (Forest et al., 2021).

Advice #5: Offer support in return when the opportunity arises.

Explanation: Mutual support is important for future offers of support and can help you to feel better and more helpful.

Specific examples:

“Feel free to let me know if I can ever help you.”

“I've heard that you're having problems with your work. Is there anything I can do to help you?”

“I have time if you need someone to talk to.”

Scientific background:

Mutual support is not just about returning favors, but also about strengthening relationships and fostering a sense of mutual caring and support. It's understandable to be hesitant to ask for help for fear of being a burden. However, research shows that people are less likely to be perceived as a burden if they express gratitude for the support they receive and reciprocate that support from time to time (Rafaeli & Gleason, 2009). So don't hesitate to ask for help when needed and thank those who offer you their support. Remember that mutual support is reciprocal and benefits everyone involved.

Advice #6: Don't always ask the same person(s) for social support.

Explanation: It is important not to rely too much on one person. The more different people you ask for support, the easier it will be for each person to take the time to be there for you. You can also benefit from the different perspectives and experiences of different people.

People who could support you well are people who recognize your support needs, your goals and your preferences for appropriate support and are motivated to help you.

Specific examples:

“Today I'm not going to ask my partner for support, but one of my friends.”

“If you are having difficulties at work, you can turn to someone from your circle of colleagues or friends who has been in a similar situation, in addition to your work colleagues.”

Scientific background:

Even if your partner or family members are your primary source of support, relying solely on them can be a significant strain on their well-being (Chai et al., 2018; Wellman, 1992). To avoid straining your relationships and ensure your own well-being, diversify your support network by seeking help from a wider range of support resources. Considering who qualified support providers might be (Rafaeli & Gleason, 2009) can help you think beyond familiar patterns of support.

We hope this advice helps you to ask [person] NOW if he/she could help with [type].

Reasons for non Adoption: Qualitative Content Analysis

To gain insights into reasons for not adopting the intervention, we conducted a qualitative content analysis of participant responses [89]. As an initial step, a team of two researchers reviewed all responses to the item sequence: *“Did you seek social support because of the app’s notification?”* If participants answered *no*, they were prompted with an open-ended question: *“Is there a reason why you did not seek social support?”* The researchers then analyzed these responses to identify and derive an inductively derived set of categories describing the reasons provided. Then, each response was coded into up to three of these categories. Subsequently, these categories were grouped into “main categories” in accordance with qualitative content analysis methodology [89].

Category Frequencies

Table S2 presents the categories and main categories as well as the frequency of occurrence for each category and summed per main category. Since responses could be coded into multiple categories, the total number of coded instances (*n* = 249) exceeds the number of unique responses (*n* = 214).

Table S5

Frequency of Categories and Main Categories as Reasons Provided for Non Adoption

Main Category	Category	Category Frequency	Main Category Frequency

Occupied (self)	No time / No capacity (e.g. too busy)	29	
	Work self	29	
	Wrong moment	21	79
No need	No need	31	
	Mood good	6	
	Already knew Cue or Cue wasn’t helpful	1	
	No guidance from app wanted / Annoyance with app	1	
	Problem/ Feelings dissolved	1	40
Internal barriers	No motivation to seek help	21	
	Mental hurdle too high	12	
	Problem too intimate/personal	2	35
Occupied (others)	No available person	9	

	Not the right person available	9	
	Work others	5	
	Person not reached	4	27
External barriers	Inappropriate	8	
	Nobody seen as appropriate for this problem	5	
	Too late (Night)	3	
	No opportunity	2	
	Quarrel with preferred person	1	19
Other forms of coping	Need for retreat / Retreat preferred	8	
	Already coped independently	5	
	Independent Coping preferred	3	
	Seeked help from animal	1	17

too sick/tired	Too tired / No energy	6	
	Mood too bad	2	
	Unwell (physically)	2	10
Coping successful	Already asked for help / arranged meetup or call	4	
	Already received help	2	6
Other	Not identifiable	14	
	Forgot	1	
	Vacation	1	16

Participants provided 214 unique reasons for not seeking social support following app notifications, resulting in 249 coded instances across multiple categories. The most frequently reported main category was "Occupied (self)" (*n* = 79), primarily due to participants having no time or capacity (*n* = 29) or work-related constraints (*n* = 29). The next most common reason was "No need" (*n* = 40), where participants felt no current need for support. Internal psychological barriers (*n* = 35), including low motivation or mental hurdles, also significantly impacted

support-seeking. "Occupied (others)" (*n* = 27) reflected the unavailability of suitable support persons, while "External barriers" (*n* = 19) involved situational constraints such as timing and appropriateness. Other less frequent reasons included alternative coping strategies ("Other forms of coping," *n* = 17), health-related limitations ("Too sick/tired," *n* = 10), and previously successful coping (*n* = 6).

References

Barbee, A. P., & Cunningham, M. R. (1995). An Experimental Approach to Social Support Communications: Interactive Coping in Close Relationships. *Annals of the International Communication Association*, *18*(1), 381–413. https://doi.org/10.1080/23808985.1995.11678921

Brans, K., Van Mechelen, I., Rimé, B., & Verduyn, P. (2014). To share, or not to share? Examining the emotional consequences of social sharing in the case of anger and sadness. *Emotion*, *14*(6), 1062–1071. https://doi.org/10.1037/a0037604

Chai, Y. C., Mahadevan, R., Ng, C. G., Chan, L. F., & Md Dai, F. (2018). Caregiver depression: The contributing role of depression in patients, stigma, social support and religiosity. *International Journal of Social Psychiatry*, *64*(6), 578–588. https://doi.org/10.1177/0020764018792585

Cutrona, C. E., & Russell, D. W. (1990). Type of social support and specific stress: Toward a theory of optimal matching. *Social support: An interactional view.*, *January*, 319–366.

Forest, A. L., Walsh, R. M., & Krueger, K. L. (2021). Facilitating and motivating support: How support-seekers can affect the support they receive in times of distress. *Social and Personality Psychology Compass*, *15*(6), 1–21. https://doi.org/10.1111/spc3.12600

Lee, D. S., Orvell, A., Briskin, J., Shrapnell, T., Gelman, S. A., Ayduk, O., Ybarra, O., & Kross, E. (2020). When chatting about negative experiences helps-and when it hurts: Distinguishing adaptive versus maladaptive social support in computer-mediated communication. *Emotion (Washington, D.C.)*, *20*(3), 368–375. https://doi.org/10.1037/emo0000555

Marshall, E. M., Karantzas, G. C., Romano, D., Lee, J., Feeney, J. A., Mullins, E. R., McCabe, M. P., & Simpson, J. A. (2023). Older adults' support seeking from their adult children: The Support-Seeking Strategy Scale. *Journal of Family Psychology*, *37*(6), 841–852. https://doi.org/10.1037/fam0001063

Pauw, L. S., Sauter, D. A., Van Kleef, G. A., & Fischer, A. H. (2018). Sense or sensibility? Social sharers' evaluations of socio-affective vs. cognitive support in response to negative emotions. *Cognition and Emotion*, *32*(6), 1247–1264. https://doi.org/10.1080/02699931.2017.1400949

Pearlin, L. I., & McCall, M. E. (1990). Occupational Stress and Marital Support. In J. Eckenrode & S. Gore (Hrsg.), *Stress Between Work and Family* (S. 39–60). Springer US. https://doi.org/10.1007/978-1-4899-2097-3_3

Rafaeli, E., & Gleason, M. E. J. (2009). Skilled Support Within Intimate Relationships. *Journal of Family Theory & Review*, *1*(1), 20–37. https://doi.org/10.1111/j.1756-2589.2009.00003.x

Rains, S. A., Ashtaputre, A., Nemcova, K., Lutovsky, B. R., Tsetsi, E., Pavlich, C. A., & Akers, C. (2021). The evolution of supportive conversations: Tracking within-discussion changes in support seeking and provision messages. *Communication Monographs*, *88*(4), 483–505. https://doi.org/10.1080/03637751.2021.1889009

Rimé, B. (2009). Emotion elicits the social sharing of emotion: Theory and empirical review. *Emotion Review*, *1*(1), 60–85. https://doi.org/10.1177/1754073908097189

Schat, E., Tuerlinckx, F., Smit, A. C., de Ketelaere, B., & Ceulemans, E. (2021). Detecting Mean Changes in Experience Sampling Data in Real Time: A Comparison of Univariate and Multivariate Statistical Process Control Methods. *Psychological Methods*. https://doi.org/10.1037/met0000447

Schwarzer, R., & Luszczynska, A. (2008). How to Overcome Health-Compromising Behaviors. *European Psychologist*, *13*(2), 141–151. https://doi.org/10.1027/1016-9040.13.2.141

Walsh, R. M., & Forest, A. L. (2021). Can Expressing Positivity Elicit Support for Negative Events? A Process Model and Review. *Personality and Social Psychology Review: An Official Journal of the Society for Personality and Social Psychology, Inc*, *25*(1), 3–40. https://doi.org/10.1177/1088868320961899

Wellman, B. (1992). Which Ties Provide What Kinds of Support? *Advances in Group Processes*, *9*, 207–235.

Williams, S. L., & Mickelson, K. D. (2008). A paradox of support seeking and rejection among the stigmatized. *Personal Relationships*, *15*(4), 493–509. https://doi.org/10.1111/j.1475-6811.2008.00212.x
